# A 96-well culture platform enables longitudinal analyses of engineered human skeletal muscle microtissue strength

**DOI:** 10.1038/s41598-020-62837-8

**Published:** 2020-04-24

**Authors:** Mohammad E. Afshar, Haben Y. Abraha, Mohsen A. Bakooshli, Sadegh Davoudi, Nimalan Thavandiran, Kayee Tung, Henry Ahn, Howard J. Ginsberg, Peter W. Zandstra, Penney M. Gilbert

**Affiliations:** 1 Institute of Biomaterials and Biomedical Engineering, University of Toronto, Toronto, Canada; 2 Donnelly Centre for Cellular and Biomolecular Research, Toronto, Canada; 3 Li Ka Shing Knowledge Institute, St. Michael’s Hospital, Toronto, Canada; 4 Department of Surgery, University of Toronto, Toronto, Canada; 5 Michael Smith Laboratories and the School of Biomedical Engineering, University of British Columbia, Vancouver, Canada; 6 Department of Biochemistry, University of Toronto, Toronto, Canada; 7 Department of Cell and Systems Biology, University of Toronto, Toronto, Canada

**Keywords:** Musculoskeletal models, Tissue engineering, Biomedical engineering

## Abstract

Three-dimensional (3D) *in vitro* models of human skeletal muscle mimic aspects of native tissue structure and function, thereby providing a promising system for disease modeling, drug discovery or pre-clinical validation, and toxicity testing. Widespread adoption of this research approach is hindered by the lack of easy-to-use platforms that are simple to fabricate and that yield arrays of human skeletal muscle micro-tissues (hMMTs) in culture with reproducible physiological responses that can be assayed non-invasively. Here, we describe a design and methods to generate a reusable mold to fabricate a 96-well platform, referred to as MyoTACTIC, that enables bulk production of 3D hMMTs. All 96-wells and all well features are cast in a single step from the reusable mold. Non-invasive calcium transient and contractile force measurements are performed on hMMTs directly in MyoTACTIC, and unbiased force analysis occurs by a custom automated algorithm, allowing for longitudinal studies of function. Characterizations of MyoTACTIC and resulting hMMTs confirms the capability of the device to support formation of hMMTs that recapitulate biological responses. We show that hMMT contractile force mirrors expected responses to compounds shown by others to decrease (dexamethasone, cerivastatin) or increase (IGF-1) skeletal muscle strength. Since MyoTACTIC supports hMMT long-term culture, we evaluated direct influences of pancreatic cancer chemotherapeutics agents on contraction competent human skeletal muscle myotubes. A single application of a clinically relevant dose of Irinotecan decreased hMMT contractile force generation, while clear effects on myotube atrophy were observed histologically only at a higher dose. This suggests an off-target effect that may contribute to cancer associated muscle wasting, and highlights the value of the MyoTACTIC platform to non-invasively predict modulators of human skeletal muscle function.

## Introduction

Skeletal muscle is one of the most abundant tissues in the human body and it enables critical physiological and functional activities, such as thermogenesis^1^ and mobility^2^. There are many degenerative and fatal diseases of skeletal muscle that remain untreated and the underlying pathology of some muscle related diseases is not fully understood. The use of animal models to study skeletal muscle diseases has improved our understanding of *in vivo* drug response and disease pathology^3,4^. However, in some cases animal models fail to accurately predict drug response in humans, in part due to species specific differences leading to inaccurate disease symptoms^5,6^. Furthermore, animal models are expensive and time consuming making them less desirable for drug testing^7^. As a result, a push to establish *in vitro* models of human skeletal muscle with reliable phenotypic readouts for drug testing is underway with the goal of improving therapeutic outcomes in humans.

Two-dimensional (2D) cultures of human skeletal muscle cells are most often implemented for drug testing and disease modeling. Despite their ease of use and demonstrated predictive power in certain cases^8^, 2D models of skeletal muscle are ill-suited for *in vitro* studies of contractile myotubes^9^ by failing to maintain structural integrity over long periods of time^10,11^, and yielding randomly oriented myotubes which limits their application for contractile force measurement^12–15^. A recent report combined 2D culture substrate micropatterning to more closely mimic the physiological environment, increase reproducibility, and provide an indirect method to assess the contractile capacity of myotubes^16^. This advancement enables scalability and high throughput drug discovery predictions. However, the method is limited in its capacity to maintain myotubes long-term, reflected in screens designed to evaluate drugs effects on the earliest phase of differentiation, and analysis is an end-point, thereby precluding longitudinal studies.

Three-dimensional (3D) tissue engineering methods to study skeletal muscle in a dish serve to address these 2D culture gaps, and are beginning to replace conventional assay platforms^17,18^. 3D culture models provide multi-dimensional cell-matrix interactions, which is critical to the pathology of conditions such as muscular dystrophies and age-induced muscle fibrosis^19,20^. In addition, engineered 3D skeletal muscle models mimic native muscle architecture^21^, provide structural integrity for long-term culture of myotubes *in vitro*, and enable contractile force measurements^22^. Recent articles report successful development of 3D culture models of human skeletal muscle^9,23–33^. In these studies, active force is quantified on tissues removed from the supporting culture device to implement a force transducer, which is precise, but invasive.

As a non-invasive alternative, others have engineered elegant culture devices that employ flexible posts or pillars to support tissue maturation and measure active force as it relates to post-deflection following electrical or chemical stimulation^17,18,27,28,30–32,34–39^. However, some of these technologies have footprints that are not amenable to high-throughput tests. In other cases, device fabrication and/or implementation is perceived as challenging, or they make use of cumbersome inserts with vertical posts, all potentially limiting their widespread adoption for drug testing and disease modeling by industry and other researchers.

Here, we describe a method for simple fabrication of a human skeletal muscle (Myo) microTissue Array deviCe To Investigate forCe (MyoTACTIC); a 96-well plate platform for bulk production of human muscle microtissues (hMMTs) for phenotypic drug testing. Our fabrication methodology leads to the reproducible single-step casting of a 96 well plate that offers easy workflow integration and requires a relatively small number of cells for tissue formation. We provide an optimized method for formation of functional hMMTs using primary human myogenic progenitor cells in MyoTACTIC by building upon previously published protocols^9,24^. We show that hMMTs self-organize in MyoTACTIC to form multi-nucleated, striated myotubes that are responsive to electrical and biochemical stimuli with kinetics and maturation levels matching those observed in larger formats^24^, and that the process is reproducible from well-to-well of the device.

MyoTACTIC enables easy and non-invasive contractile force and calcium transient measurements of hMMTs over time within the culture device (*in situ*). We demonstrate that known myotoxic drugs (dexamethasone, cerivastatin) induce myotube atrophy and decrease hMMT contractile force generation similar to clinical outcomes, while treating hMMTs with insulin-like growth factor 1 (IGF-1) improves contractile force generation. We then show that a single clinically relevant dose of Irinotecan, a chemotherapeutic reagent used to treat pancreatic cancer, induces myotube atrophy and diminishes contractile force, thereby validating the ability of MyoTACTIC to predict an off-target drug response on human skeletal muscle.

We focused our studies on direct myotube effects by initiating all treatments at a time-point when the myotubes are fully formed, an assessment made possible by the MyoTACTIC system. Notably, we uncover modified force responses at lower treatment doses than those required to see visible effects by classic histological methods, highlighting the sensitivity of functional metrics over end-point morphological assessments.

In sum, MyoTACTIC enables longitudinal analyses of human skeletal muscle microtissue strength to support fundamental research, drug discovery, and toxicity studies.

## Results

### MyoTACTIC fabrication and implementation is simple and supports bulk production of human skeletal muscle micro-tissues

**Figure 1:**
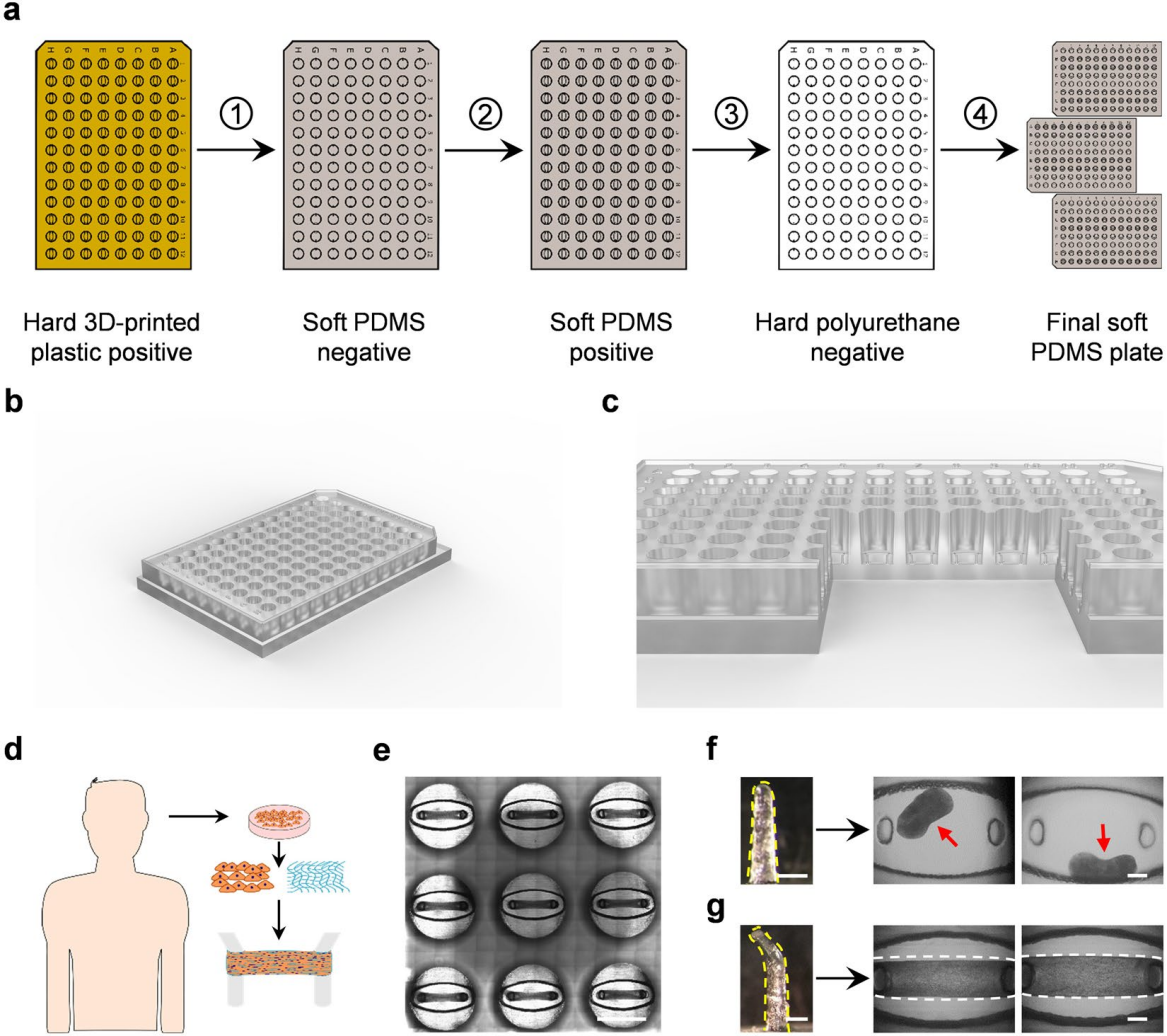
Design and production of the MyoTACTIC platform. (**a**) MyoTACTIC production started with creating a three-dimensional Computer Aided Design (3D CAD) using SolidWorks™ Software which was then printed using a ProJet MJP 3500 3D printer from 3D SYSTEMS. Next, a negative PDMS mold was created from the 3D printed part which was subsequently used to fabricate a soft replica of the design after silanizing. Finally, a rigid negative polyurethane mold was created from the PDMS replica which was used to fabricate multiple PDMS plates. **(b,c)** Computer generated 3D images of **(b)** MyoTACTIC 96-well plate design and **(c)** a cross-section of wells indicating the location of the micro-posts. **(d)** Schematic overview of human cell isolation and subsequent generation of hMMTs in MyoTACTIC. **(e)** Stitched phase-contrast image of 9 wells of MyoTACTIC containing remodeled hMMTs 10 days post seeding. Scale bar 5 mm. **(f,g)** Impact of micro-post design on formation and long-term maintenance of hMMTs in MyoTACTIC. Representative images of **(f)** collapsed and **(g)** successfully remodeled hMMTs seeded in wells with **(f)** hook-less and **(g)** hook featured posts. Micro-posts are outlined in yellow dashed lines. Red arrows indicate collapsed hMMTs on the top right panels and hMMTs are outlined in white dashed lines on the bottom right panels. Scale bars 500 µm.

We report an *in vitro* platform, hereafter referred to as MyoTACTIC, that supports simple and reproducible culture of contractile human skeletal muscle microtissues (hMMTs) for drug and biomolecule testing. MyoTACTIC is a custom-designed elastomeric 96-well plate (Fig. 1a–c and Supplementary Fig. 1) in which each well consists of an elliptical inner chamber containing two vertical posts at either end (Fig. 1c,e–g and Supplementary Fig. 1). A multi-step casting process is employed to fabricate MyoTACTIC plates (Fig. 1a) from a 3D printed design (see Materials and Methods). The fabrication process leads to the generation of a reusable polyurethane (PU) negative mold for reproducible generation of MyoTACTIC plates containing up to 96-wells and all well features using single step polydimethylsiloxane (PDMS) casting within 3 hours (Fig. 1a, Step 4).

**Figure 2:**
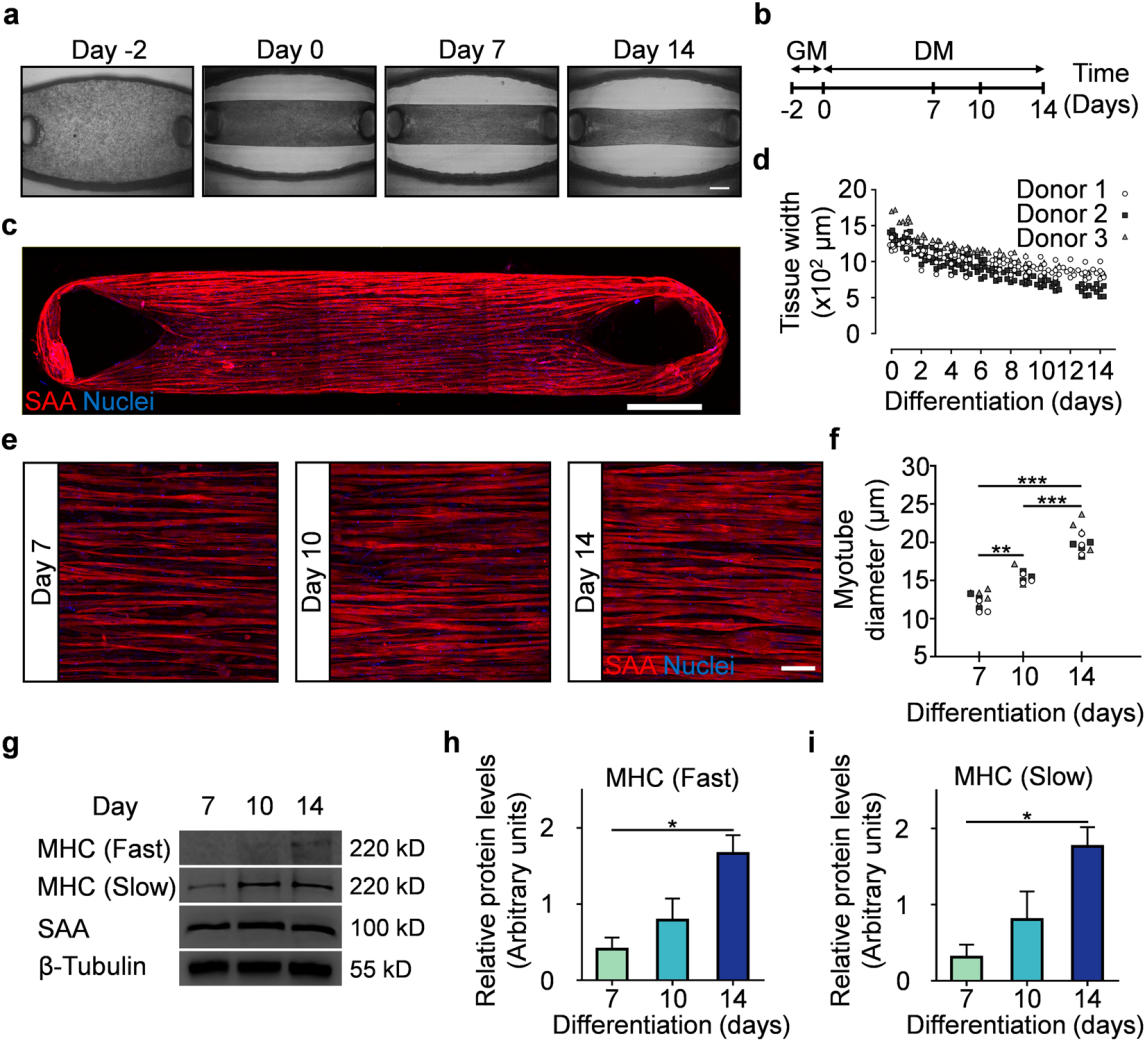
MyoTACTIC supports formation of hMMTs with aligned myotubes exhibiting hypertrophy and adult myosin heavy chain expression. (**a**) Representative phase-contrast images of hMMTs depicting the remodeling of the extracellular matrix by human myoblasts over time. Day 0 marks the time for switching to differentiation media. Scale bar 500 µm. **(b)** Schematic diagram of the timeline for hMMT culture. hMMTs are cultured in growth media (GM) lacking bFGF for the first two days (day -2 to day 0) and then the media is switched to differentiation media (DM) on Day 0. **(c)** Representative confocal stitched image of a hMMT cultured for 2 weeks, immunostained for sarcomeric α-actinin (SAA, red) and exposed to DRAQ5 (blue) to counter stain the nuclei. Scale bar 500 µm. **(d)** Dot plot indicating the width of hMMTs over the course of culture time. (n = minimum of 16 hMMTs from 3 muscle patient donors per time point). **(e)** Representative confocal images of myotubes formed in hMMTs and immunostained for SAA (red) and nuclei (blue) after 7, 10, and 14 days in differentiation media. Scale bar 50 µm. **(f)** Quantification of hMMT myotube diameter over time. **p < 0.01, ***p < 0.001 (n = minimum of 9 hMMTs from 3 muscle patient donors per time point). (**g**) Representative western blot images of myosin heavy chain (MHC) isoforms (fast and slow), SAA, and β-tubulin over culture time (Day 7, 10, and 14). The blots were cropped and stained separately (see Methods for more detail). Full length blots are presented in Supplementary Fig. 8. (**h–i**) Bar graph quantification of relative (**h**) MHC-fast and (**i**) MHC-slow protein expression in hMMTs over culture time. *p < 0.05 (n = 3 blots from 3 muscle patient donors, where each blot was run in a set of single experiment using lysate of 4 hMMTs (per time point) from a single patient donor lysed together. Blots were then processed in parallel to generate the bar graphs shown in (**h**,**i**)). In (**d**,**f**) each symbol represents data from one patient donor. In (**h**,**i**) values are reported as mean ± SEM. In **(f**,**h**,**i)** significance was determined by one-way ANOVA followed by multiple comparisons to compare differences between groups using Tukey’s multiple comparisons test.

Three-dimensional (3D) hMMTs were engineered using purified primary human myogenic progenitor cells (Fig. 1d and Supplementary Fig. 2a) suspended in a hydrogel mix (Table S2) based on previously published work^9,24^, by pipetting the cell-hydrogel suspension into the MyoTACTIC well chambers, between and around the posts (Fig. 1c–e and Fig. 2a**, left panel**). The micro-posts included in each well serve as tendon-like anchor points to establish uniaxial tension in the remodeling hMMT (Fig. 1c,e–g) and direct micro-tissue formation and compaction in each well (Fig. 1c,e,g and Supplemental Fig. 2b).The hook feature at the top of each post is an essential design criteria as hMMTs migrate off of hook-less posts within two days of cell seeding (Fig. 1f). The bottom of the slanted tips of each micro-post marks the height where each hMMT is positioned after remodeling (Supplementary Fig. 3a). Importantly, we observed consistent migration of hMMTs to the position below the slanted tip of micro-posts within 7 days in differentiation inducing culture media (Supplementary Fig. 3a,b). The hMMTs remained at this location for at least one additional week (Supplementary Fig. 3a,b). Indeed, MyoTACTIC design (e.g. post size, shape, and positioning, cell seeding chamber size and shape, platform material stiffness, etc.) was iterated to enable bulk production of hMMT arrays (Supplementary Fig. 4) amenable to the ‘in dish’ functional analyses described herein.

### Human muscle microtissues cultured in MyoTACTIC exhibit structural characteristics similar to macro- tissues

In order to ensure that hMMTs cultured in MyoTACTIC possess structural characteristics comparable to previously published methods^9,24^, in spite of using fewer cells and a miniaturized format, we investigated the structural characteristics of hMMTs in culture over time. hMMTs remodeled within two days of culture in myogenic growth media without bFGF (Fig. 2a,b and Table S2) and continued to remodel and compact over the additional two weeks in differentiation media (Fig. 2a,b,d and Supplementary Fig. 5a).

Within two weeks of differentiation, hMMTs formed multi-nucleated and aligned myotubes as evident by sarcomeric α-actinin (SAA) immunofluorescence analysis (Fig. 2c). The vast majority of cells were post-mitotic by Day 7 of differentiation (Supplementary Fig. 5b), and we also identified formation of cross-striated myotubes containing clustered acetylcholine receptors (AChRs) at this time point, which persisted until Day 14 (Supplementary Fig. 5c). We quantified myotube width at Days 7, 10 and 14 of differentiation (Fig. 2e,f). As expected, myotube width increased with time in culture. We noted that hMMT myotube hypertrophy is sensitive to and supported by autocrine cues, underlying the importance of reserving a portion of the hMMT conditioned media during media exchanges (Supplementary Fig. 5d). Finally, structural maturation of the hMMTs in culture was evident by significantly higher expression of the adult subtypes (fast and slow^40^) of the contractile protein myosin heavy chain (MHC) at the later stages of culture time (Fig. 2g–i), while SAA^24^ protein expression remained relatively steady over time (Fig. 2g).

Our analysis demonstrates that hMMTs cultured in MyoTACTIC exhibit similar remodeling dynamics, structural characteristics, and maturation levels to their macroscale counterparts^9,24^. Further, while some variation is observed between biological replicates, variation amongst technical replicates is negligible (Fig. 2d,f), hence we conclude that the process of hMMT development within MyoTACTIC is reproducible.

### MyoTACTIC allows for non-invasive and *in situ* hMMT contractile force assessment

hMMTs cultured in MyoTACTIC exhibited spontaneous contractions in culture between Days 10 to 12 of differentiation (Supplementary Movie S1), and were competent to produce twitch and tetanus contractions in response to electrical stimuli (0.5 Hz and 20 Hz respectively; Supplementary Movie S2 and Supplementary Fig. 6) at this time-point. As predicted based on the presence of AChR clusters (Supplementary Fig. 5c), hMMTs generated an immediate and robust tetanus contraction in response to biochemical (ACh, 2 mM) stimulation (Supplementary Movie S3). These observations confirm hMMT functional maturation in MyoTACTIC.

**Figure 3:**
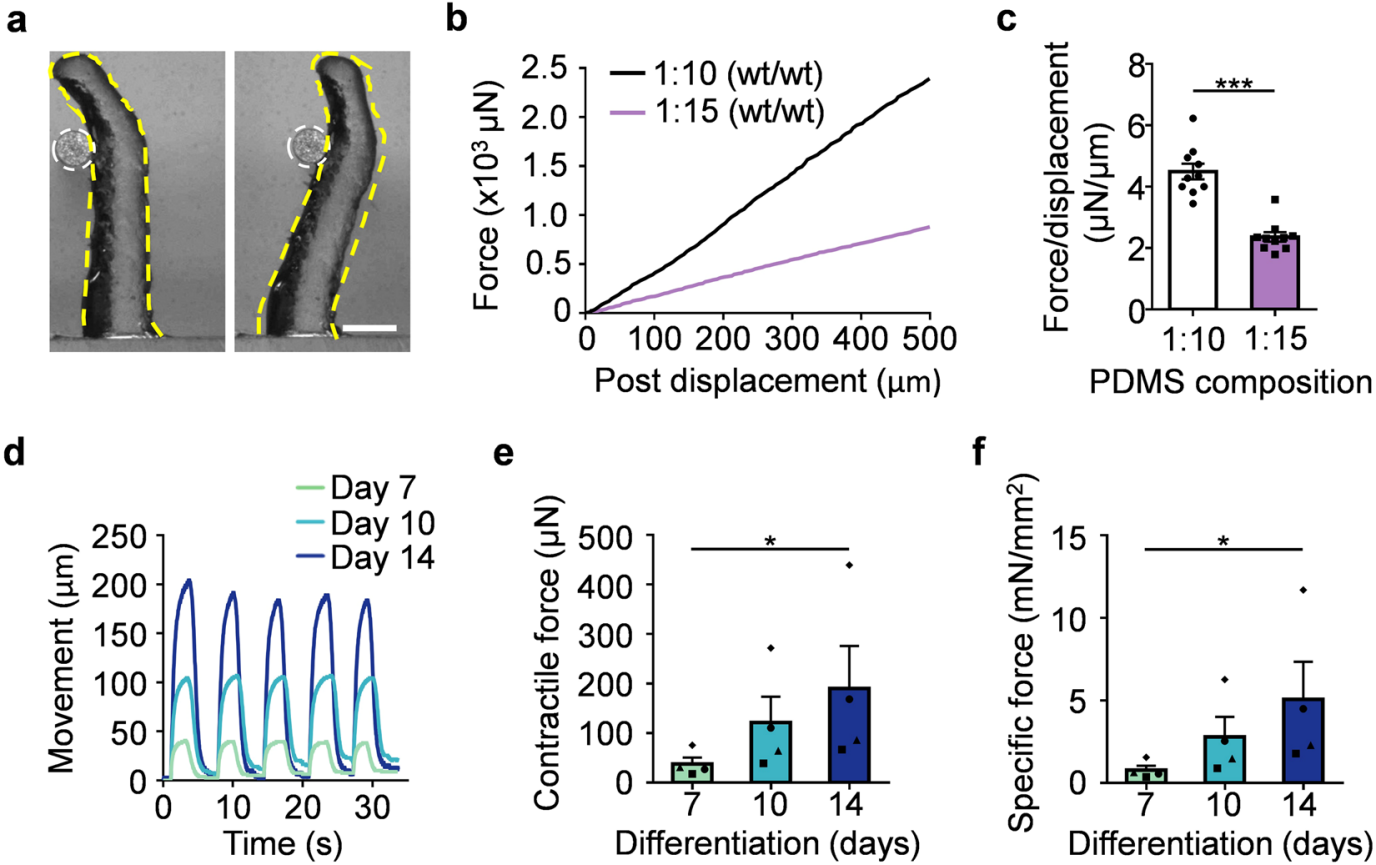
MyoTACTIC enables non-invasive and *in situ* measurement of hMMT contractile force. (**a**) Phase-contrast images of a micro-post (outlined in yellow dashed lines) displaced in response to the force exerted by a microwire (outlined in white dashed circles). Scale bar 500 µm. **(b)** Plot depicting the relation between force and displacement of micro-posts fabricated using two different PDMS compositions (curing agent weight (wt): monomer weight), as measured by the Microsquisher. **(c)** Bar graph quantification of the average force/displacement ratio for the micro-posts formed using two different PDMS compositions. ***p < 0.001 (n = 10 micro-posts per condition). **(d)** Representative line graph traces of the micro-post displacement during high frequency (20 Hz) electrical stimulation of hMMTs at Day 7, 10, and 14 of differentiation measured by the custom-written Python computer vision script. **(e**,**f)** Bar graph quantification of the absolute **(e)** and specific **(f)** tetanus contractile forces generated by hMMTs at Day 7, 10, and 14 of differentiation. *p < 0.05 (n = minimum of 11 hMMTs from 4 muscle patient donors per time point). In **(e**,**f)** each symbol represents averaged hMMT data from one patient donor. In **(c**,**e**,**f)** values are reported as mean ± SEM. Significance was determined by t-test in **(c)** or Friedman test followed by Dunn’s multiple comparisons test to compare differences between groups in **(e**,**f)**.

*In vitro* models of human skeletal muscle are generally incapable of studying muscle contraction, and often those that can are limited to doing so as an experimental endpoint owing to the need to remove tissues from the culture device so as to implement a force transducer for measurements^16,24^. MyoTACTIC micro-posts, as with a recently reported cell culture insert approach^35^, were designed to sustain hMMT long-term culture and to allow for non-invasive contractile force measurements of hMMTs in an easy and robust manner (Fig. 3a). Mechanical analysis of MyoTACTIC micro-posts confirmed a linear force-displacement relation (Fig. 3a,b) in saline (37 °C). This material property allows for hMMT contractile force to be determined by comparing the extent of post deflection^17^ induced by hMMTs during contraction in response to electrical and biochemical stimuli (Fig. 3a,b).

Since the mechanical modulus of the micro-post design can be modified by adjusting the PDMS monomer to curing agent ratio (Fig. 3c), it is possible to tune micro-post deflection properties (Fig. 3b). To maximize post deflection, which in turn serves to minimize measurement error, we implemented the lower curing agent to monomer ratio (1:15 wt/wt) for the force measurements in this study. Notably, well-to well-variation in micro-post mechanical properties was minimal (Fig. 3c). Next, we developed a post tracking script in Python to non-invasively, and *in situ*, quantify hMMT strength through analysis of post deflection videos (Supplementary Fig. 7a, Supplementary Movie S4, and **Appendix**). Using the semi-automated and unbiased post tracking script, we confirmed that the absolute and specific tetanus contractile forces of the hMMTs increases significantly with culture time from Day 7 to 14 of differentiation (Fig. 3d–f and Supplementary Movie S5). Force magnitudes were in line with prior reports of human skeletal muscle microphysiological systems at similar culture timepoints^24,35^. While a noticeable variation in the magnitude of the contractile forces generated by hMMTs was observed between each muscle donor at reported culture time points, a significant increase in contractile forces with culture time (Days 7–14) was observed consistently within each muscle donor (Fig. 3e,f). The same could be observed when contractile forces were normalized to the Day 7 values (Supplementary Fig. 7b).

Here we conclude that MyoTACTIC, together with our post tracking script, provides a powerful system for longitudinal phenotypic studies of hMMT force generation.

### MyoTACTIC enables non-invasive and *in situ* hMMT calcium transient assessment

**Figure 4:**
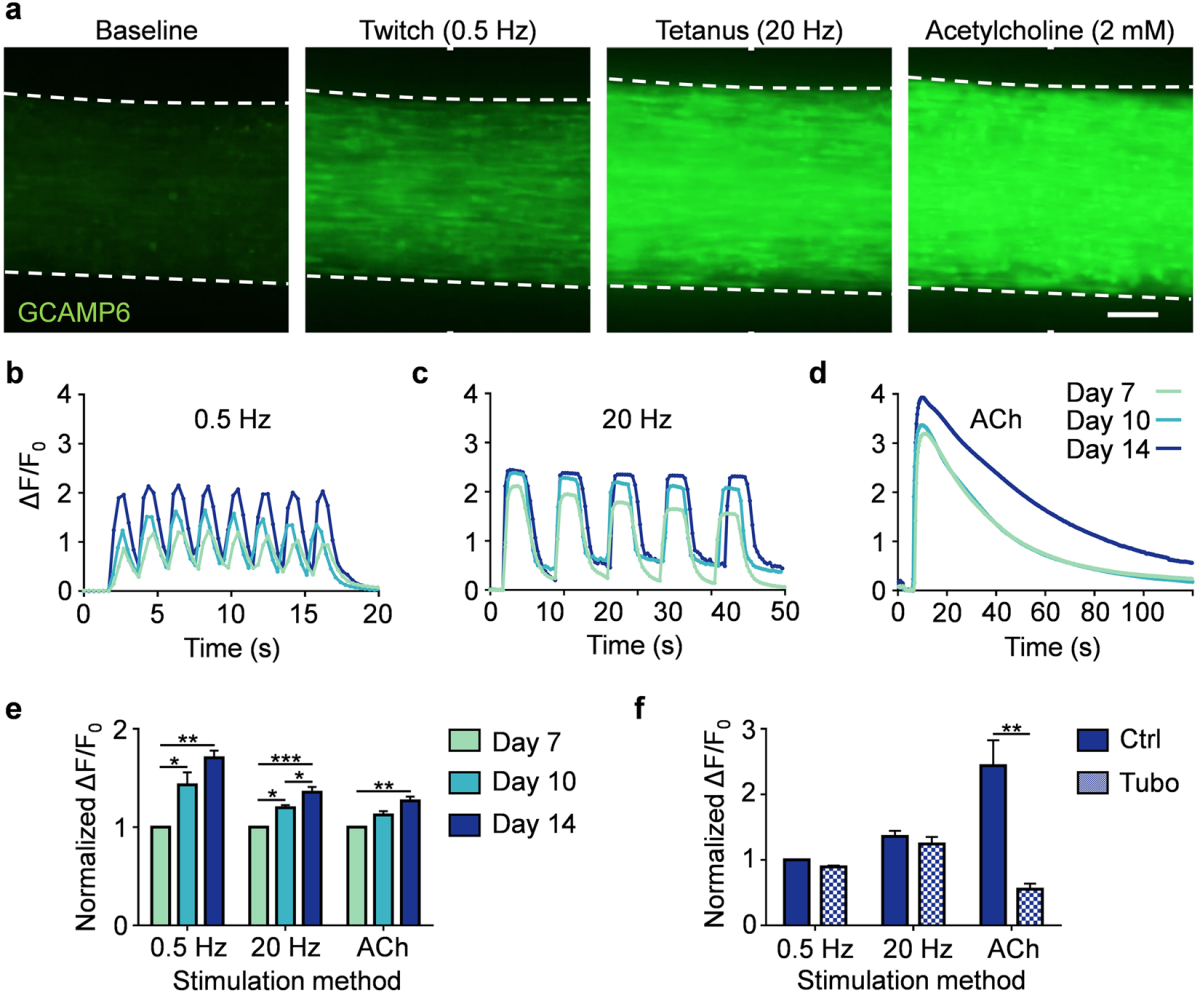
MyoTACTIC enables non-invasive and *in situ* measurement of hMMT calcium transients. (**a**) Representative epifluorescence images of the peak GCaMP6 signal from hMMTs in response to low frequency (0.5 Hz, twitch contraction), high frequency (20 Hz, tetanus contraction) and acetylcholine (ACh, 2 mM) stimulations at Day 14 of differentiation. Scale bar 200 µm. **(b–d)** Representative calcium transient traces of hMMTs at Day 7, 10, and 14 of differentiation in response to **(b)** low and **(c)** high frequency electrical and **(d)** acetylcholine stimulations. **(e)** Bar graph quantification of hMMTs calcium transients in response to electrical (0.5 and 20 Hz) and biochemical (ACh) stimuli at differentiation Day 7, 10 and 14. Values are normalized to the Day 7 results for each stimulation modality. *p < 0.05; **p < 0.01, ***p < 0.001 (n = minimum of 9 hMMTs from 3 muscle patient donors per time point, per stimulation method). **(f)** Bar graph quantification of calcium transients in hMMTs activated with electrical or biochemical stimuli following pre-treatment with d-tubocurarine (25 µM) at Day 14 of differentiation. Values are normalized to control (Ctrl) hMMTs stimulated with 0.5 Hz electrical stimuli. **p < 0.01 (n = 9 hMMTs from 3 muscle patient donors per treatment condition per stimulation method). In **(e**,**f)** values are reported as mean ± SEM. Significance was determined by two-way ANOVA followed by Tukey’s and Sidak’s multiple comparisons to compare differences between groups in **(e)** or t-test in **(f)**.

To evaluate the calcium handling properties of hMMTs in MyoTACTIC, we generated microtissues using GCaMP6f stably transduced human muscle progenitor cells^41^, a sensitive calcium indicator protein, driven by the MHCK7^24^ promoter, a muscle specific gene. hMMTs exhibited spontaneous myotube calcium transients (Supplementary Movie S6) in as early as Day 7 of differentiation. GCaMP6 signals arising from hMMTs are captured in the context of MyoTACTIC, allowing for assessment over time. hMMTs generated strong collective calcium transient in response to electrical stimulation (Fig. 4a–c and Supplementary Movies S7) and immediately following exposure to ACh (Fig. 4a,d and Supplementary Movie S8). The magnitude of stimulated calcium transients increased significantly with culture time as measured by normalized fluorescence intensity (∆F/F_0_; Fig. 4b–e). Furthermore, pre-treatment with d-tubocurarine (25 µM) blocked ACh-induced calcium transients (Fig. 4f and Supplementary Movie S9), but had no significant effect on hMMT calcium transients induced by electrical stimuli (Fig. 4f and Supplementary Movie S9), which mimics the *in vivo* muscle response^42,43^, and validates the functional activity of AChR clusters formed on hMMTs (Supplemental Fig. 5c).

### MyoTACTIC cultured hMMTs predict structural and functional treatment responses

**Figure 5:**
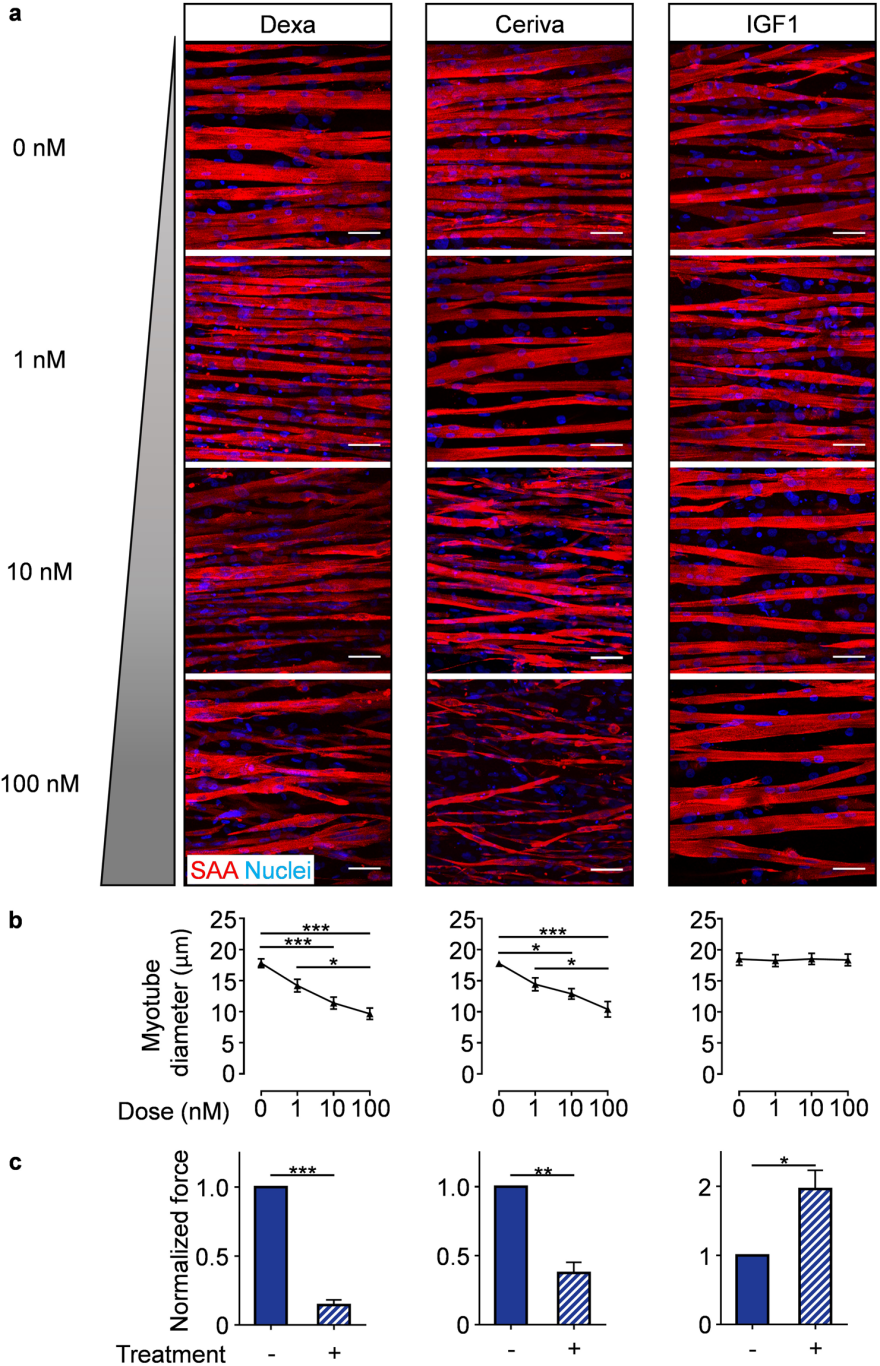
MyoTACTIC-cultured hMMTs predict skeletal muscle structural and functional responses to pharmacological treatment. (**a**) Representative confocal images of Day 14 hMMTs treated for 7 days with either vehicle control or increasing doses (1, 10, and 100 nM) of Dexamethasone (left panels), Cerivastatin (middle panels), or IGF-1 (right panels) and immunostained for sarcomeric α-actinin (SAA, red) and Hoechst 33342 (Nuclei, blue). Scale bar 50 µm. **(b)** Quantification of the dose-dependent effect of Dexamethasone (left panel), Cerivastatin (middle panel), and IGF-1 (right panel) on hMMT myotube diameter. *p < 0.05, ***p < 0.001 (for each treatment, n = minimum of 4 hMMTs from a minimum of 3 muscle patient donors per treatment dose). (**c**) Bar graph quantification of the tetanus (20 Hz electrical stimuli) contractile force generated by hMMTs treated from Day 7 to 14 with either vehicle (−) or (+) Dexamethasone (10 nM; left panel), Cerivastatin (10 nM; middle panel), and IGF-1 (100 nM; right panel). Ethanol, ddH_2_O, and 10 mM HCl were vehicle controls for Dexamethasone, Cerivastatin, and IGF-1 respectively. Values are normalized to vehicle-treated results on differentiation Day 14. *p < 0.05, ***p < 0.001 (for each treatment, n = minimum of 8 hMMTs generated from 3 muscle patient donors per treatment condition). In (**b,c**) values are reported as mean ± SEM. In (**b**), significance was determined by one-way ANOVA followed by Tukey’s multiple comparisons to compare differences between groups (Dexamethasone and Cerivastatin) or Kruskal Wallis test followed by Dunn’s multiple comparisons to compare differences between groups (IGF-1). In (**c**), significance was determined by t-test (Dexamethasone and Cerivastatin) or t-test with Welch’s correction (IGF-1).

Accurate drug response prediction is a crucial requirement if engineered tissues are to be implemented for drug testing. Hence, we studied hMMT myotube size and contractile strength responses to treatment with three compounds with well-studied effects on skeletal muscle. Since MyoTACTIC supports long-term culture, in this study compounds were administered from Day 7 to 14 of culture to evaluate treatment effects on multinucleated myotube morphology and function. Immunofluorescence analysis confirmed that dexamethasone and cerivastatin treatments, which are known to induce rhabdomyolysis in humans^44–46^, elicited a dose-dependent decrease in myotube width (Fig. 5a,b**, left** and **middle panels**). In contrast, hMMTs treated with IGF-1 displayed no change in myotube size, regardless of dose (Fig. 5a,b**, right panels**).

We then studied compound treatment effects on function by assessing hMMT contractile force. As predicted by morphological assessment, dexamethasone and cerivastatin treatments (10 nM) induced contractile weakness (Fig. 5c**, left** and **middle panels** and Supplementary Movies S10, S11). Notably, IGF-1 treated (100 nM) hMMTs exhibited significant contractile force gain compared to control (Fig. 5c **right panel** and Supplementary Movie S12) akin to *in vivo* animal studies^47,48^, and suggesting that MyoTACTIC is capable of perceiving compound effects through non-invasive force analysis that might otherwise be overlooked using more conventional methods^49^.

To ensure that the changes in the contractile forces were not due to a change in the positioning of hMMTs on the micro-posts following drug treatment, we assessed the location of hMMTs on Day 7 (pre-treatment) and Day 14 (post-treatment). Our analysis revealed no significant changes in the position of hMMTs on the micro-posts in response to treatments that strengthen (IGF-1, 100 nM) or weaken (dexamethasone, 100 nM) the hMMTs (Supplementary Fig. 3c–f).

Together, our data demonstrate that hMMT treatments in MyoTACTIC accurately reflect the known effects and can be evaluated via a non-invasive metric of function.

### MyoTACTIC study predicts direct effect of chemotherapeutic drug on human skeletal muscle

Given the predictive response of hMMTs to treatments with known effects on skeletal muscle, we next applied the system to interrogate potential skeletal muscle off-target effects of cancer chemotherapeutic drugs. Cancer-induced skeletal muscle wasting, known as cachexia, has a poorly understood pathology. Cachexia is emerging as an independent determinant of patient survival^50,51^, suggesting treatment strategies designed with skeletal muscle health in mind are desirable. Since cancer cells are characterized in part by deregulated proliferation, many commonly used chemotherapeutic agents non-selectively target cycling cells. Skeletal muscle fibers are post-mitotic, hence, a direct effect of chemotherapeutic agents on muscle health is not expected and therefore underexplored. Patients with pancreatic cancer are strongly associated with cachexia^50,52,53^, therefore we selected Gemcitabine and Irinotecan for MyoTACTIC analysis, both of which employ mitosis-targeting mechanisms of action^54,55^, and are used to treat patients with advanced pancreatic cancer.

**Figure 6:**
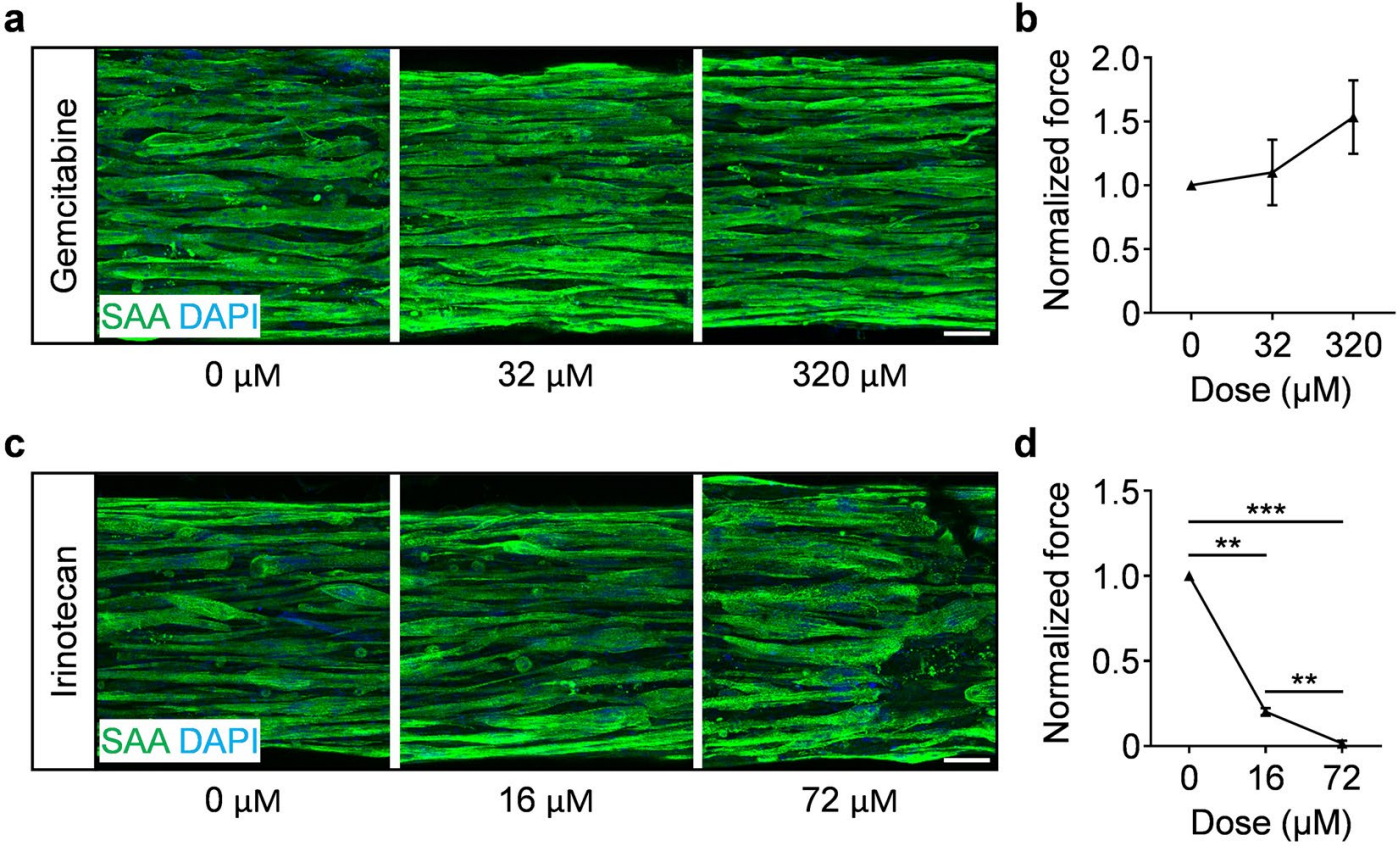
MyoTACTIC cultured hMMTs predict direct effect of chemotherapeutic agents on human skeletal muscle contractile function. (**a,c**) Representative confocal images of Day 14 hMMTs treated with one-time administration of vehicle (DMSO for Irinotecan and PBS for Gemcitabine) or dosed with **(a)** Gemcitabine or **(c)** Irinotecan on Day 7 and then immunostained for sarcomeric α-actinin on Day 14. Scale bar 50 μm. **(b**,**d)** Quantification of tetanus (20 Hz electrical stimuli) contractile force generation by Day 14 hMMTs treated with vehicle (DMSO for Irinotecan and PBS for Gemcitabine), **(b)** Gemcitabine (32 μM and 320 μM), or **(d)** Irinotecan (16 μM and 72 μM). Values in **(b**,**d)** are normalized to vehicle treated results on differentiation day 14. **p < 0.01, ***p < 0.001 (n = minimum of 4 hMMTs from 3 biological replicates per treatment condition). In (**b**,**d**) values are reported as mean ± SEM. Significance was determined by one-way ANOVA followed by Tukey’s multiple comparisons to compare differences between groups in (**b,d**).

Drug doses were selected based on reported clinical concentrations^56,57^, in addition to evaluating a supraphysiological dose for each drug. The treatment began on Day 7 of differentiation, when multinucleated myotubes are prevalent (Fig. 2) and functional (Figs. 3 and 4**)**, and few mitotic cells remain (Supplementary Fig. 5b). The regimen in this study was a single dose to model a typical clinical treatment, and followed by analysis one week later (Day 14). Gemcitabine, regardless of dose (32 µM and 320 µM), had no significant effect on hMMT myotube structural organization (Fig. 6a) or contractile force generation (Fig. 6b and Supplementary Movie S13). Conversely, Irinotecan exposure elicited a dramatic reduction in hMMT strength at the clinical dose (16 µM) with clear effects on myotube integrity only visible histologically at the supraphysiological dose (Fig. 6c,d and Supplementary Movie S14**)**.

This study extolls the capacity of MyoTACTIC to predict unexpected off-target effects on skeletal muscle force generation with a greater sensitivity than standard histological methods.

## Discussion

Here we report a design and methods to fabricate a 96-well culture platform, MyoTACTIC, for the bulk production of hMMTs. We show the feasibility of hMMT for phenotypic compound testing using an image based readout in a moderate throughput fashion. Each well of MyoTACTIC contains a cell seeding chamber and two vertical, flexible micro-posts that support hMMT self-organization and whose movement is tracked in short videos to measure hMMT force *in situ*. We used a combination of 3D printing and microfabrication techniques to produce a reusable PU mold that can be employed to cast a large number of PDMS devices (>100) in a single step. Single step casting eliminates the perceived challenge or technical issues arising in the use of reported culture platforms or inserts containing vertical posts^17,18,25,35,58,59^. The MyoTACTIC platform makes hMMT production fast, reproducible, and user-friendly.

As mentioned, a notable advantage of MyoTACTIC is its capacity to quantify hMMT active force non-invasively and *in situ*, in a 96-well format, by using post deflection captured in videos coupled with our Python tracking script. The non-invasive nature of our readouts is of critical importance since it enables longitudinal studies of drug effects on hMMTs at different time points. This challenge is faced by currently available methods where assessment of 3D tissue passive force^58^, stimulated 2D culture contractility^16^, or stimulated 3D tissue force^24,60^ are implemented as endpoint assays. While there are certainly examples of 3D culture platforms that enable force measurement based on flexible-post deflection and image analysis^17,18,27,35,38^, we expect that the combined 96-well footprint, one step fabrication of a stand-alone device, and ease of use will be key elements to promote widespread adoption by other researchers. While, we show moderate throughput for tissue seeding and data capture, future investigations can take advantage of the standard 96-well plate footprint of MyoTACTIC to couple it with 3D bioprinting and automated liquid handling stations to improve the throughput of hMMT formation and culture. In addition, smartphone applications can be designed to measure the force of hMMTs while capturing tissue contractions (micro-post deflections). This will facilitate image based readouts to further increase the throughput of the system making it more suitable for larger screens.

While 3D printing is an excellent technique for product prototyping due to its speed and cost-effectiveness, the technology still faces limitations with regards to the resolution of the technique for microfabrication. As seen in our Supplementary Data (Supplementary Fig. 3g), we observed the presence of uneven surfaces behind each post in our first printing trials, which prevented hMMTs from moving upward to the intended position just below the slanted region. We hypothesized that this was due to the 3D printing resin which remained intact despite extensive wax removal efforts. Consistently, in subsequent iterations, modifying the printing direction improved the uneven surfaces and resulted in consistent placement of hMMTs at the expected location on the posts, immediately below the slanted part of the micro-posts from differentiation Day 7 onward (Supplementary Fig. 3a–f). Therefore, given the reproducibility of micro-post mechanical measurements obtained across wells from multiple MyoTACTIC devices (Fig. 3c), we are confident in the capability of our device to quantify changes in hMMT contractile force.

Another point to consider is the damage to a subset of posts that occurs during fabrication Steps 1 to 3 (Fig. 1a), which can lead to the formation of unusable wells in the hard PU negative mold. We hypothesize that this is due to the sharp angles required for the final portion of plate demolding. Table S4 details the number of intact micro-posts in the original 3D printed 96-well plate we received from the 3D printing facility. We then observed an ~ 10% reduction in the number of functional wells in the resulting PU molds. However, after fabrication of the PU negative mold, Step 4 (Fig. 1a) routinely yields many rounds of PDMS casting with no damage to the remaining wells (Table S4). This validates the fitness of PU as the material of choice for PDMS microfabrication. To achieve defect-free MyoTACTIC devices, an alternative method to fabricate the final product after the prototyping stage, such as micromachining, could be employed to address the 3D printing shortcomings noted above.

We next conducted studies to confirm that the hMMTs cultured in MyoTACTIC exhibit similar structural and functional characteristics reported in previously published work^9,24^. In this regard, we show that MyoTACTIC enables the formation of hMMTs that display comparable myotube hypertrophy, adult contractile protein expression, and calcium handling trends as reported in larger engineered human skeletal muscle tissue formats (Figs. 2, 4)^9,24^. Consistently, hMMTs cultured in MyoTACTIC exhibited formation of cross-striated myotubes, in as early as 7 days of differentiation, that contained clustered AChRs and responded to biochemical (ACh) and electrical stimuli by contracting (Figs. 2–4 and Supplementary Fig. 5c). Using GCaMP6 transduced human skeletal muscle progenitor cells and capitalizing on the transparency of the MyoTACTIC plate, we recorded hMMT calcium transients *in situ* and over time from Day 7 to 14 and observed the maturation process (Fig. 4).

To validate the application of MyoTACTIC for drug testing, we first focused our studies on compounds with well-studied effects on human skeletal muscle^44–46,61^. An advantage of MyoTACTIC over previously reported drug testing culture systems is the ability to support long-term culture. Hence, we focused all of our biological studies on compound administration regimes that started on Day 7 of culture when myotubes were competent to produce force (Fig. 3) and few mononucleated cells remained (Supplementary Fig. 5b), and assessed hMMTs one week later (Day 14). As expected, dexamethasone and cerivastatin induced myotube atrophy, as observed in histological analysis, and reduced hMMT contractile force generation, in a dose-dependent manner (Fig. 5).

We next turned our attention to demonstrating the capacity of MyoTACTIC to detect compounds that increase strength. Studies in animals^47,48,62^ support a role for IGF-1 in muscle hypertrophy. Culture studies in which IGF-1 is delivered to mono-nucleated myogenic progenitors when exposed to differentiation conditions in 2D culture^16,63,64^, or to nascent myotubes in 2D^65,66^ or 3D^17^ culture also observed hypertrophic effects by immunohistological analysis. Interestingly, we did not find a change in the width of IGF-1 treated hMMT myotubes even at a dose reported by others to induce myotube hypertrophy^66^. We expect this is explained by a difference in treatment regime, with other studies initiating IGF-1 treatment regimens at earlier stages of differentiation. Regardless, we observed a significant increase in the force generated by IGF-1 treated hMMTs, which might be attributed to IGF-1 effects on skeletal myotube protein translation^66,67^. Indeed, visual inspection of IGF-1 treated hMMTs reveals a uniformity in myotube width along the entire structure hinting at contractile apparatus maturation. This signifies the predictive value of the MyoTACTIC platform in capturing drug and biomolecule effects using a clinically relevant functional readout for skeletal muscle. A point to consider is the probable adsorption of peptides and small molecules by PDMS due to hydrophobic interactions. However, albumin which is available in serum is known to passively bind to PDMS^68^ and minimizes excessive adsorption of peptides to the PDMS surface^69^. Given that our culture media is rich in serum (20% FBS in growth media and 2% horse serum in differentiation media) and our drug treatment initiates on Day 7, the surface adsorption of the drugs by PDMS in our system is of a lesser concern. However, a permanent coating is required to prevent biomolecule adsorption to the PDMS surface in the context of serum free culture studies in MyoTACTIC^70–72^.

hMMT studies offer the unique opportunity to decouple direct and indirect effects of systemic treatments on skeletal muscle health. To highlight this point, we sought to interrogate potential direct influences of chemotherapeutic agents on hMMT morphology and strength (Fig. 6). Cachexia, a specific type of muscle wasting, is frequently described in cancer patients, including those with pancreatic cancer^50,52,53^, and it is associated with decreased life expectancy^51^. The cause of cancer-associated cachexia is a very active area of research.

In general, the direct effect of cancer chemotherapeutic agents on skeletal muscle health is understudied, since most are developed with a mitosis-targeting mechanism of action, and skeletal muscle fibers are post-mitotic. However, evidence suggested that human skeletal muscle might be a direct target of two mitosis-targeting chemotherapeutic drugs; Gemcitabine and Irinotecan. First, a study in healthy mice evaluated skeletal muscle following systemic treatment with Folfox or Folfiri, two commonly implemented pancreatic cancer patient chemotherapy cocktails. Loss of muscle mass and weakness were observed in animals treated with Folfiri and not Folfox, with Irinotecan being the variable discerning the two cocktails, though this point was not explored in the report^73^. Interestingly, a study of mouse C2C12 cells in culture noted that Irinotecan induced changes in cycling myoblast mitochondrial activity, but treatment had little influence on myotube width^74^. We implemented MyoTACTIC to study the direct effects of these widely used chemotherapeutic agents on hMMT function. Strikingly, a one-time treatment of a clinical dose of Irinotecan (16 µM^56^ on Day 7) led to a significant decrease in hMMT contractile force as compared to carrier control treatment. Most notably, histologically apparent effects on myotube integrity were only uncovered at a higher dose of Irinotecan (72 µM), highlighting the sensitivity of our non-invasive functional assay. Even the highest dose of Gemcitabine produced no detectable effect on myotube morphology or strength. Together, this data provides motivation to conduct epidemiological studies in this area with the goal of informing chemotherapeutic regimes tailored to the patient as their needs evolve over the course of treatment.

## Methods

### Platform fabrication for human muscle micro-tissue (hMMT) culture

Fabrication of the final polydimethylsiloxane (PDMS) platform (MyoTACTIC) was accomplished through sequential fabrication of intermediate plates as shown in Fig. 1a. The original MyoTACTIC template was designed in SOLIDWORKS (Supplementary Fig. 1) and 3D-printed using ProJet MJP 3500 Series from 3D SYSTEMS. VisiJet M3 Crystal was used as the printing material. The template was then cast into a flexible PDMS plate using the Sylgard 184 silicone elastomer kit (Dow Corning) (Fig. 1a, step 1). During casting, the template was lightly sprayed with Ease Release® 200 release agent (Smooth-On) and left in a chemical hood for 10 to 15 minutes to ensure consistent spread of the release agent. Liquid PDMS (10 parts monomer to one part curing agent (weight/weight)) was degassed in a vacuum chamber and poured onto the original 3D-printed template. A container was used to keep the liquid PDMS in place. Next, the container (including the template and the liquid PDMS) was degassed in a vacuum chamber for 45 minutes to remove any bubbles trapped within the PDMS. The container was then placed in an isothermal oven set to 60 °C overnight. The next day the cured negative PDMS mold was manually separated from the template. The negative PDMS plate was then used as a template to cast a positive PDMS plate (Fig. 1a, step 2). To ensure the release of the second PDMS part from the negative PDMS mold during this PDMS to PDMS casting step, a surface modification was applied to the template as previously described^75^. Briefly, the negative PDMS mold was plasma-treated for 2 minutes in a plasma chamber (Harrick Plasma). Plasma-treated negative PDMS mold was immediately coated with 1 H, 1 H, 2 H, 2H-perfluorodecyltrichlorosilane (Sigma) in a vacuum chamber for at least 3 hours. Next, liquid PDMS (10 parts monomer to one part curing agent) was poured onto the silane-coated PDMS negative. To promote the penetration of liquid PDMS into the small features, the negative mold was placed inside a vacuum chamber and thorough degassing was performed for at least one hour with intermittent vacuum breaks. Next the container was placed in an isothermal oven at 60 °C overnight. The cured positive PDMS mold was then separated from the negative PDMS mold. Next, the positive PDMS mold was used as a template to fabricate a negative polyurethane (PU) plate (Fig. 1a, step 3). To cast the negative PU plate, the positive PDMS plate was sprayed with a light layer of Ease Release® 200 and left at room temperature in a chemical safety hood for at least 15 minutes to ensure even spread of the release agent. Next, Smooth-Cast® 310 liquid plastic (Smooth-On) was prepared based on the manufacturer’s instructions and was poured on top of the positive PDMS mold in a container and left at room temperature until fully solidified (minimum of 3 hours). Subsequently the PDMS plate was separated from the PU mold. At this stage, the rigid PU negative mold enabled one-step fabrication of the MyoTACTIC platform. Copies of MyoTACTIC were then produced by repeatedly using the PU negative mold as a template (Fig. 1a, step 4). The PU template was sprayed with a light layer of Ease Release® 200 and liquid PDMS (15 parts monomer to one part curing agent (weight/weight)) was poured onto the PU mold. Next, the mold was degassed thoroughly in a vacuum chamber to ensure the total penetration of PDMS into the small features of the PU mold. The construct was placed in an isothermal oven at 60 °C for at least 3 hours, after which the final PDMS device, MyoTACTIC, (Fig. 1b) was separated from the negative PU mold.

### Quantification of micro-post mechanical properties

Mechanical properties of the micro-posts were determined using a Microsquisher device (CellScale). Briefly, micro-posts with their PDMS base were excised from each well of a MyoTACTIC using a sharp blade and mounted in the Microsquisher test chamber, immersed in 37 °C PBS. A micro-wire (Fig. 3a) connected to a force transducer was used to deflect micro-posts with known amounts of force (Supplementary Movie S15), and quantified the extent of micro-post displacement that was induced. In this way, Microsquisher analysis quantified the relationship between the magnitude of force applied to micro-posts, and their displacement (Fig. 3b,c).

### Human skeletal muscle biopsy material

The collection and use of human skeletal muscle tissue was reviewed and approved by the Providence St. Joseph’s and St. Michael’s Healthcare Research Ethics Board (REB# 13-370) and the University of Toronto Office of Research Ethics reviewed the approved study and further assigned administrative approval (Protocol# 30754). Informed consent was obtained from all study participants. All methods in this study were performed in accordance with the guidelines and regulations of these two Research Ethics Boards. Human skeletal muscle tissues removed during scheduled surgical procedures and designated for disposal were utilized in this study. Small skeletal muscle samples (~1 cm^3^) were obtained from the multifidus muscle of patients undergoing lumbar spine surgery. Participant information is provided in Table S5.

### Primary human myoblast preparation and expansion

Primary human myoblast cell lines were established and maintained as previously described^9^. Briefly, human skeletal muscle samples were minced and then dissociated into a single cell slurry with Clostridium histolyticum collagenase (Sigma, 630 U/mL) and dispase (Roche, 0.03 U/mL) in Dulbecco’s Modified Eagle’s medium (DMEM; Gibco). The cell suspension was passed multiple times through a 20 G needle to facilitate the release of the mononucleated cell population and subsequently depleted of red blood cells with a brief incubation in red blood cell lysis buffer (Table S2). The resulting cell suspension containing a mixed population of myoblasts and fibroblast-like cells was plated in a collagen-coated tissue culture dish containing myoblast growth medium: F-10 media (Life Technologies), 20% fetal bovine serum (Gibco), 5 ng/mL basic fibroblast growth factor (bFGF; ImmunoTools) and 1% penicillin-streptomycin (Life Technologies). After one passage, the cell culture mixture was stained with an antibody recognizing the neural cell adhesion molecule (NCAM/CD56; BD Pharmingen; Table S1), and the myogenic progenitor (CD56^+^) population was separated and purified using fluorescence-activated cell sorting (FACS) and maintained on collagen-coated dishes in the myoblast growth medium. Subsequent experiments utilized low passage cultures (P4 - P9).

### Fabrication of human muscle microtissues (hMMTs)

Human muscle microtissues were generated following our previously described method^9^. Briefly, FACS purified CD56^+^ myogenic progenitor cells were suspended in a hydrogel mixture (Table S2) at 15 ×10^6^ cells/ml. Thrombin (Sigma) was added at 0.2 to 0.5 unit per mg of fibrinogen prior to seeding the cell/hydrogel mixture in MyoTACTIC wells. Tissues were then incubated for 5 minutes at 37 °C to expedite thrombin-mediated fibrin polymerization. Myoblast growth media (Table S2) lacking bFGF, but containing 1.5 mg/mL 6-aminocaproic acid (ACA; Sigma), was added. 2 days later the growth media was exchanged to myoblast differentiation medium (Table S2) containing 2 mg/ml ACA. This time point is referred to as differentiation Day 0. Half of the culture media was exchanged every other day thereafter until the desired experimental endpoint unless otherwise indicated.

### hMMTs drug treatments

Drugs were purchased as listed in Table S3. All drugs were sterile-filtered for use before adding to the media. Dexamethasone and Cerivastatin were dissolved in ethanol and ddH_2_O at 2.548 mM and 4.352 mM respectively. Subsequently, 50X stock solutions were prepared for each dose by diluting the drug solutions in DMEM. IGF-1 was prepared in 10 mM HCl solution at 100 μM of the highest dose and diluted to 50X stock solutions for each dose using DMEM. Gemcitabine was reconstituted in PBS at 40X (12.8 mM) stock solution for its highest dose and diluted in DMEM for lower doses. Irinotecan was prepared in DMSO at 31.75 mM of its highest dose and diluted to 21X stock solutions for each dose using DMEM. Vehicle solutions (ethanol, ddH_2_O, PBS, HCl, or DMSO) were added to the culture media of control hMMTs at concentrations corresponding to the highest drug doses administered. Drug treatment of hMMTs was initiated on differentiation Day 7. To maintain the concentration of drugs in the media, Dexamethasone and Cerivastatin were added to the hMMT media every other day during media change time points, while IGF-1 was added daily at full dose. Chemotherapeutic drugs were added to the hMMT media once on differentiation Day 7. Half the media was replaced with fresh media every other day thereafter until differentiation Day 14.

### Immunostaining and fluorescence microscopy

hMMTs were fixed and labeled for confocal imaging as previously described^9^. Briefly, hMMTs were fixed on the posts in 4% PFA for 15 minutes and then washed with PBS. Following fixation, samples were incubated in blocking solution (Table S2) for 1 hour at room temperature or overnight at 4 °C. Samples were then incubated in primary antibody (Table S1) solutions diluted in blocking solution (Table S2) overnight at 4 °C. After several washes in blocking solution or PBS, samples were incubated with appropriate secondary antibodies diluted in the blocking solution for 30–60 minutes at room temperature. Hoechst 33342 or DRAQ5 (abcam) were used to counterstain cell nuclei. Confocal images were acquired with Fluoview-10 software using an Olympus IX83 inverted microscope. Phase-contrast images were acquired with CellSense^TM^ software using an Olympus IX83 microscope equipped with an Olympus DP80 dual CCD color and monochrome camera or an Apple^®^ iPhone^®^ SE. Images were analyzed and prepared for publication using NIH ImageJ software.

### Myotube size analysis

Myotube size was analyzed as previously described^9^. Briefly, myotube size was measured by assessing 20X and 40X magnification confocal images of immunostained hMMTs for sarcomeric α-actinin. Z-stack images of hMMTs were analyzed to quantify the diameter of each intact myotube at the center of each image using NIH ImageJ. Average myotube diameter was determined per image, and these values were then averaged to calculate the average myotube diameter for each hMMT.

**Remark**: A minimum of 4 confocal images were captured per hMMT at multiple locations (non-overlapping). Subsequently a minimum of 10 myotube diameters were analyzed per confocal image.

### Western blotting

hMMTs were collected at the indicated time points and immediately lysed in RIPA buffer (Thermofisher) containing Halt™ protease inhibitor cocktail (Thermofisher). Total protein concentration was measured using BCA assay kit (Thermofisher) and 20 to 25 µg of protein was run on an 8% SDS-PAGE gel. Western blot was performed as described previously^9^. Briefly, western blot was performed using a Bio-Rad Power Pac 1000 and Trans-Blot Turbo Transfer System to transfer the proteins from the polyacrylamide gel to a nitrocellulose membrane. Each gel was run in a set of single experiment using lysate from minimum of 4 hMMTs (per each time point) from a single patient donor that were pooled and lysed together. After the transfer to the nitrocellulose membrane, the blot was cropped into multiple bands to be stained and imaged separately (see Fig. 2g and Supplementary Fig. 8). Membranes (cropped blots) were incubated overnight at 4 °C with primary antibodies against beta-tubulin, sarcomeric α-actinin, myosin heavy chain slow and fast in milk-based blocking solution (Table S2) (please refer to Table S1 for the concentrations and vendors). Next, membranes were washed 3 ×30 minutes with rocking in a Tris-buffered saline with Tween (TBST; Table S2) and then transferred into blocking solution containing horseradish peroxidase conjugated anti-rabbit and anti-mouse secondary antibodies (Cell Signaling; 1:5000). Chemoluminescence was performed using ECL substrate (ThermoFischer) with MicroChemi 4.2 chemiluminescence imaging system (DNR Bio-Imaging Systems). Images were analyzed using NIH ImageJ.

**Remark**: during the course of this study overexposure of the blots was avoided and the average exposure time for each specific protein was as provided below:

- Beta-Tubulin: 5 to 10 seconds.
- Sarcomeric α-actinin (SAA): 15 to 30 seconds.
- Myosin heavy chain (MHC) slow: 30 to 60 seconds.
- Myosin heavy chain (MHC) fast: 45 to 90 seconds.

### hMMT electrical stimulation

Two electrodes (25 G × 5/8 BD™ Regular Bevel Needles) were inserted behind the posts into each MyoTACTIC well to generate an electric field parallel to the myotubes. Electrodes were sterilized using 70% ethanol before insertion into wells. Electrodes were then connected to a commercial pulse generator (Rigol DG1022U), using nickel coated copper wires (McMaster-Carr) and alligator clamps. A Rigol DS1102E digital oscilloscope was used to confirm the frequency and amplitude of the signals before connecting the pulse generator to the wires. hMMTs were stimulated using square pulses with 20% duty cycle, 5 V amplitude (electrical field strength of 10 V/cm), and 0.5 Hz and 20 Hz frequency for twitch and tetanus contractions, respectively. A graphical representation of the electrical stimulation setup is presented in Supplementary Fig. 6.

### hMMT biochemical stimulation

Acetylcholine was reconstituted to produce a 200 mM stock solution in DMEM (100×) and was diluted to the final working concentration by addition directly into each MyoTACTIC well containing hMMTs.

### hMMT calcium transient analysis

Human myogenic progenitor cells expressing the MHCK7-GCaMP6f reporter (Addgene #65042) were generated as previously described^9,24^. hMMTs expressing GCaMP6 were imaged using an Olympus IX83 microscope at different time points following differentiation. Movies were recorded at 4X magnification at 11 frames per second using an Olympus DP80 dual CCD color and monochrome camera with a FITC filter and CellSense^TM^ software. MyoTACTIC wells containing hMMTs were placed on the microscope stage equipped with temperature and gas modules to simulate physiological conditions (37 °C and 5% CO2) during data collection. A region of interest in the center of each hMMT was defined for fluorescence analysis (Supplementary Movie S16) and movies were then analyzed using NIH ImageJ software. Relative changes in fluorescence signal were measured and are reported as ΔF/F_0_ = (F_immediate_ − F_baseline_)/(F_baseline_). The relative change in fluorescence signal was plotted against time and the peak signal of 5 to 8 consecutive stimulations (contractions) were averaged for each hMMT.

### Measurement of hMMT vertical placement on micro-posts

hMMTs were generated as described above. On differentiation Day 7, MyoTACTIC wells containing hMMTs were sectioned from the side with a sharp blade to remove extra PDMS to expose the hMMTs for imaging. Next, cross-sectioned MyoTACTIC wells containing hMMTs were placed on their side into a well of a 6-well plate. Media was added to submerge the PDMS well. PDMS pieces were fixed in place with sterile metal needles to prevent floatation in the media. Images were captured using a Leica StereoZoom S9E (under 3X magnification), an Apple^®^ iPhone^®^ SE, and a LabCam™ iPhone microscope mount. Wells were returned to their upright position. This process was repeated on differentiation Day 10 and Day 14 to acquire images of each hMMT from the side to visualize the vertical placement of tissues on the micro-posts. The images were then analyzed to measure the distance of each hMMT from the base (bottom) of the micro-posts. Briefly, the upper and lower distance of each hMMT from the base of the micro-post was measured using the NIH ImageJ software. Values were then averaged to generate a single data point for each hMMT on each micro-post. Values are reported as distance of hMMTs from the base of each micro-post in micrometers.

### Measurement of hMMT contractile force

Movies of post deflection were captured using an Apple^®^ iPhone^®^ SE during electrical stimulation of hMMTs under either 4X or 10X magnification using an Olympus IX83 microscope and a LabCam™ iPhone microscope mount (Supplementary Fig. 6c). Movies were then analyzed using a custom-written Python computer vision script (described in the **Appendix**), which determined post displacement in pixels during post deflections. hMMTs were induced to contract 5 times, and the maximum post displacements were averaged to calculate the average post deflection per hMMT in pixels. Next, post displacement pixel values were converted to absolute movements in microns using a pixel to distance conversion factor for 4X or 10X magnification objectives, as appropriate, in the NIH ImageJ software. The post displacement numbers were then converted to absolute contractile forces generated by hMMTs using the force-displacement conversion factor for the 1:15 curing agent to monomer ratio of the PDMS used in MyoTACTIC fabrication (Fig. 3c). Specific forces were measured using absolute forces divided by the average cross-sectional area of the hMMTs at each corresponding culture time point (i.e. Day 7, 10, and 14). Contractile forces of drug treated hMMTs are reported as values normalized to the corresponding untreated or vehicle treated controls, as specified in the figure caption for each drug treatment.

### EdU analysis

Click-iT® EdU cell proliferation assay kit (Thermofisher) was used for cell cycle analysis. 5-ethynyl-2′-deoxyuridine (EdU) was added at 10 µM to the culture medium of hMMTs on Day 0, Day 1, or Day 7 of differentiation (Supplementary Fig. 5b). 19 hours post-EdU administration, hMMTs were fixed and stained following the user manual. Briefly, fixed hMMTs were permeabilized in 0.3% Triton X-100 solution for a minimum of 15 minutes. Next, cells were counterstained for Alexa Fluor 647 using anti-EdU reaction solution for 30 minutes at room temperature. Nuclei were counterstained with Hoechst 33342. Images were acquired using confocal microscopy as mentioned before.

### Statistical analysis

hMMT-level data such as average myotube diameter per hMMT, average ΔF/F_0_ per hMMT, and average post deflection per hMMT, constituted technical replicates. Technical replicate data from hMMTs seeded at the same time and from the same cell source (i.e. from the same biological replicate) were averaged together to calculate a single biological replicate average. All hMMT experiments were performed with a minimum of 3 biological replicates with the exception of the data presented in Supplementary Fig. 3(c–f). Experiment specific hMMT technical and biological replicate information is contained within the figure legends. Statistical testing for significance was conducted on these biological replicate averages, while technical replicate data were used to verify the parametric assumptions of residual normality and homogeneity of variance via the Shapiro-Wilk test and the Brown-Forsythe test, respectively. Technical replicate data were transformed to pass the parametric assumptions when necessary, whereupon the relevant statistical test was performed on similarly transformed biological replicate data. When transformation of data did not resolve violations of the parametric assumptions, non-parametric tests were employed. Parametric tests used included the one-way ANOVA followed by Tukey’s multiple comparison test, the independent student’s t-test, and the independent student’s t-test with Welch’s correction. Non-parametric tests used included the Kruskal-Wallis and Friedman test followed by Dunn’s multiple comparisons test. Statistical analyses were completed using GraphPad Prism 6 (La Jolla, USA).

## Supplementary information

Supplementary Information.

Supplementary Movie 1.

Supplementary Movie 2.

Supplementary Movie 3.

Supplementary Movie 4.

Supplementary Movie 5.

Supplementary Movie 6.

Supplementary Movie 7.

Supplementary Movie 8.

Supplementary Movie 9.

Supplementary Movie 10.

Supplementary Movie 11.

Supplementary Movie 12.

Supplementary Movie 13.

Supplementary Movie 14.

Supplementary Movie 15.

Supplementary Movie 16.
