# Supplementary material for: A 96-well culture platform enables longitudinal analyses of engineered human skeletal muscle microtissue strength
Source: Sci Rep. 2020 Apr 24;10:6918. doi: 10.1038/s41598-020-62837-8 (PMC7181829; doi:10.1038/s41598-020-62837-8)
Supplement: Supplementary file 1 — Supplementary Information. [file 41598_2020_62837_MOESM1_ESM.pdf]

Supplemental Information

Title

A 96-well culture platform enables longitudinal analyses of engineered human skeletal muscle microtissue strength

Authors

Mohammad E. Afshar, Haben Y. Abraha, Mohsen A. Bakooshli, Sadegh Davoudi, Nimalan Thavandiran, Kayee Tung, Henry Ahn, Howard Ginsberg, Peter W. Zandstra, Penney M. Gilbert

Supplemental Figures and Figure Legends

Supplemental Figures

Supplementary Figure 1

a

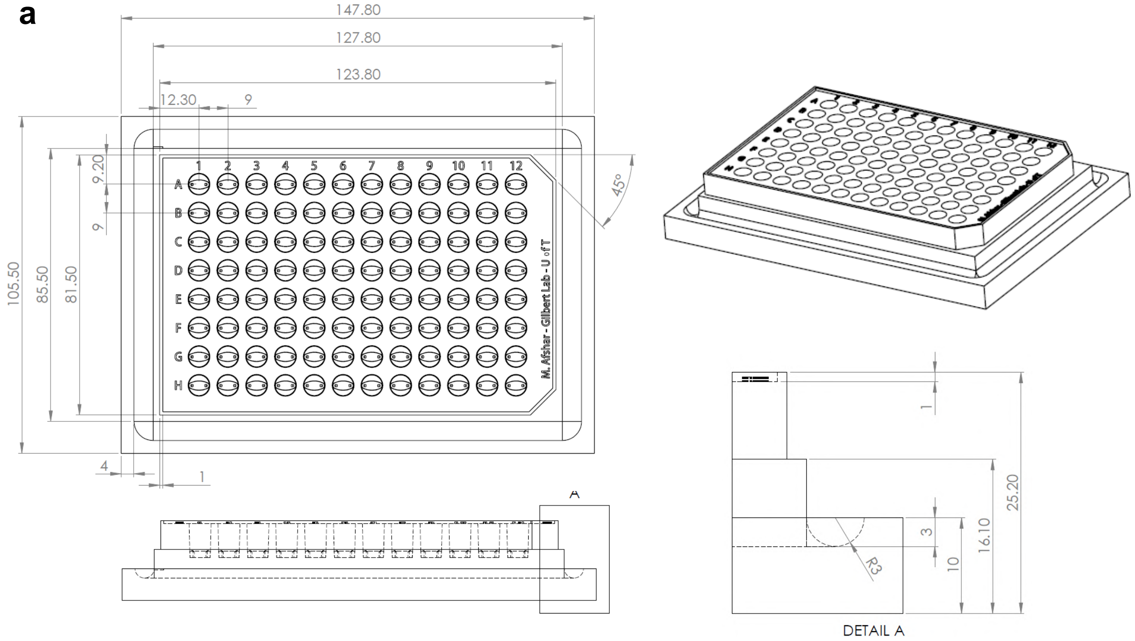

b

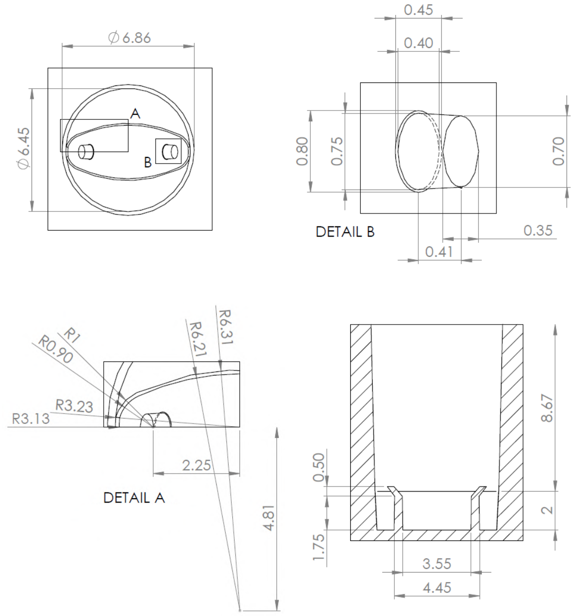

c

	Volume (mm³)	Surface Area (mm²)
Upper well (Red)	301.55	190.57
Lower well (Blue)	28.34	44.09
Posts	0.56	3.36

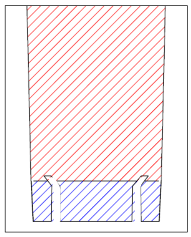

Supplementary Figure 2

a

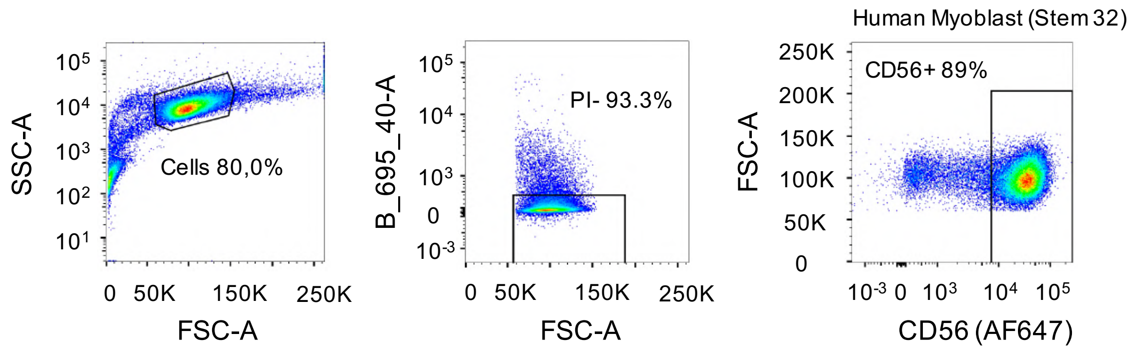

Supplementary Figure 3

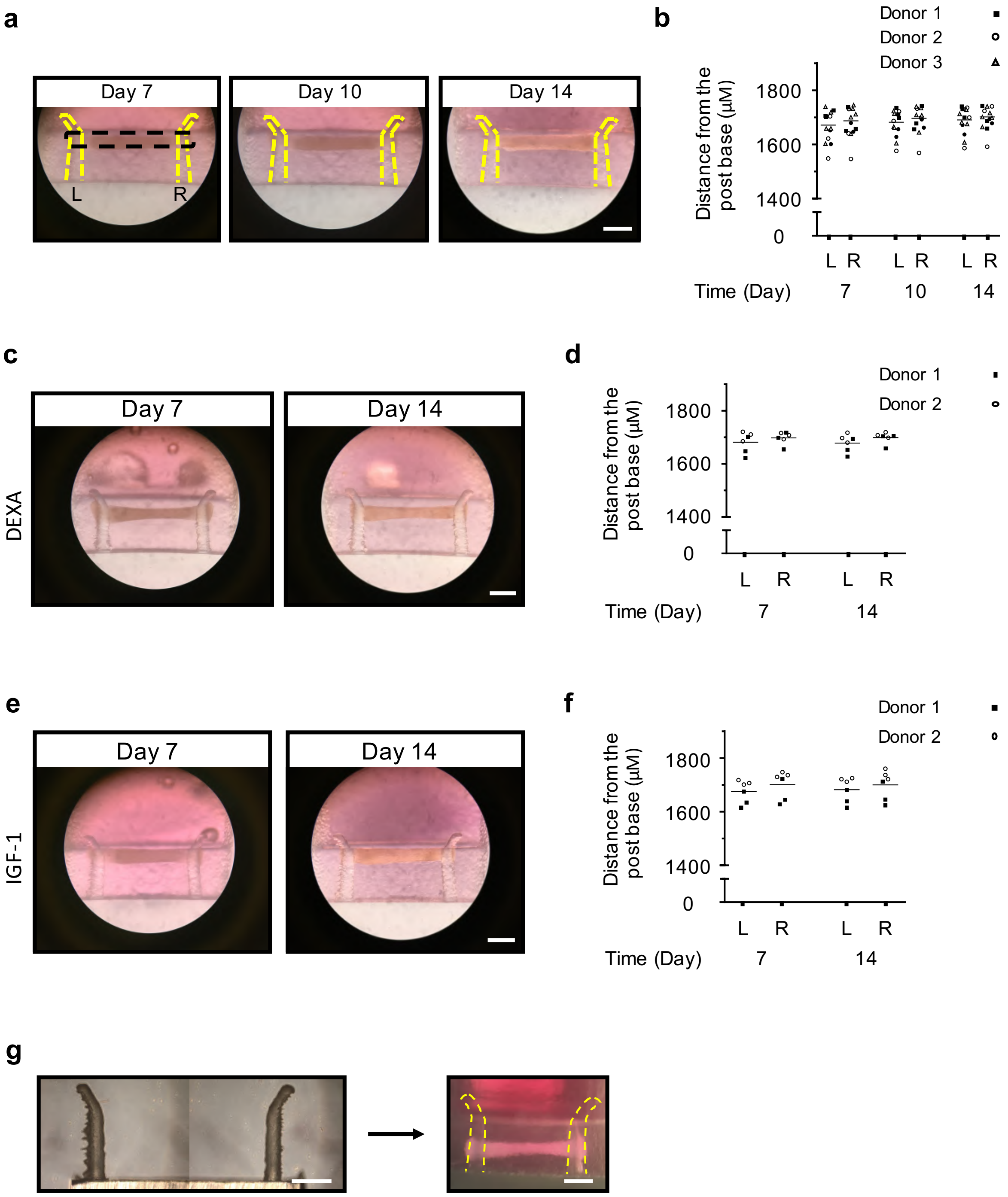

Supplementary Figure 4

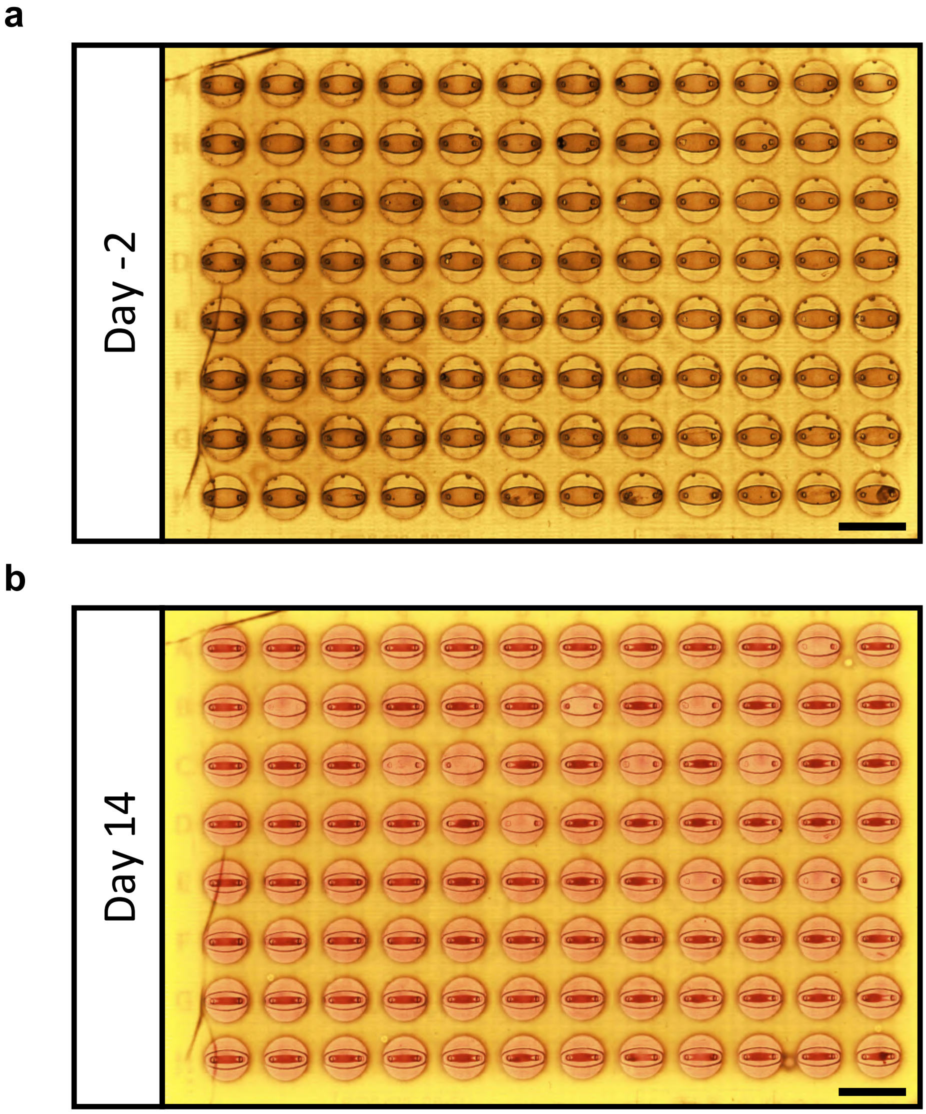

Supplementary Figure 5

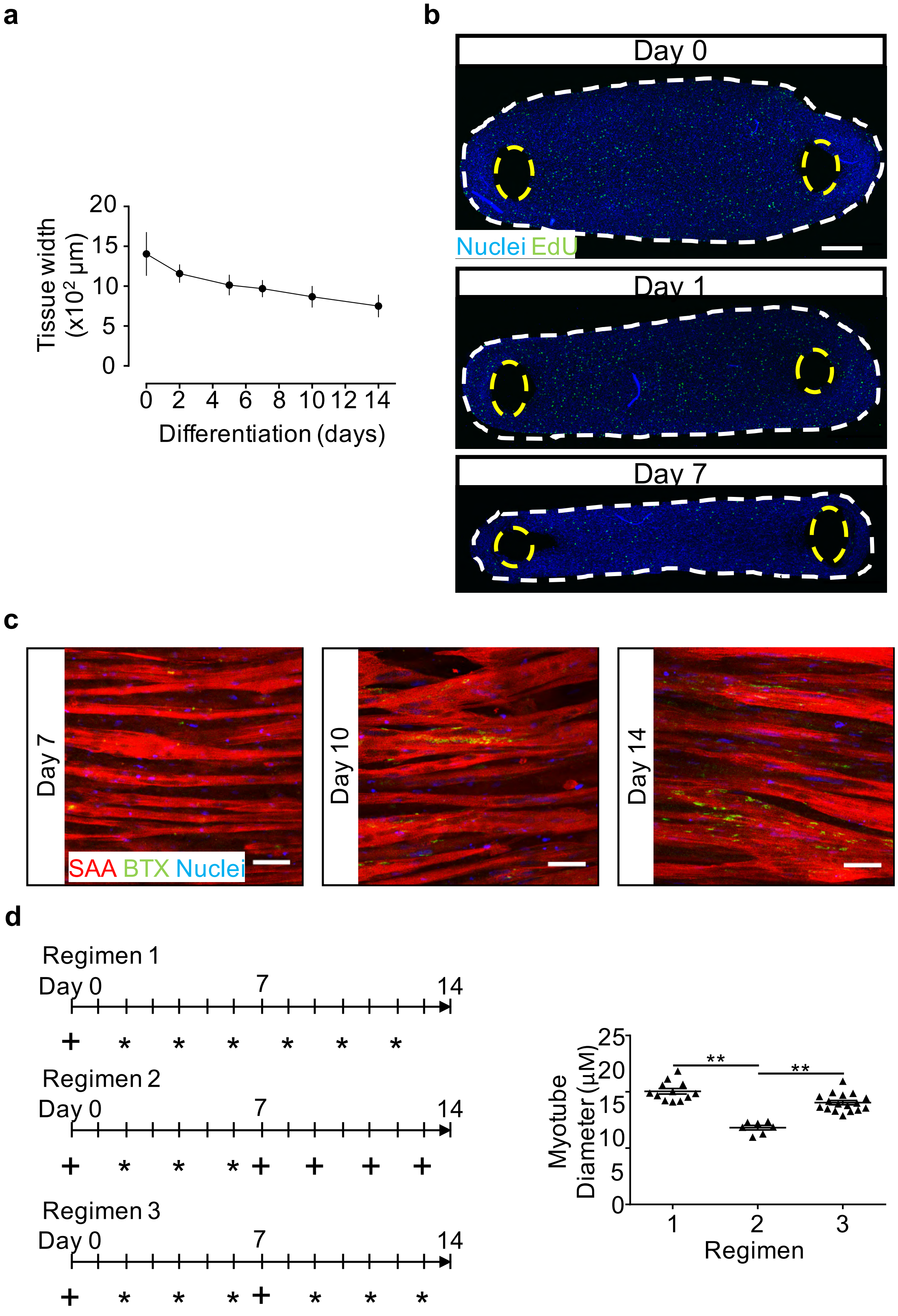

Supplementary Figure 6

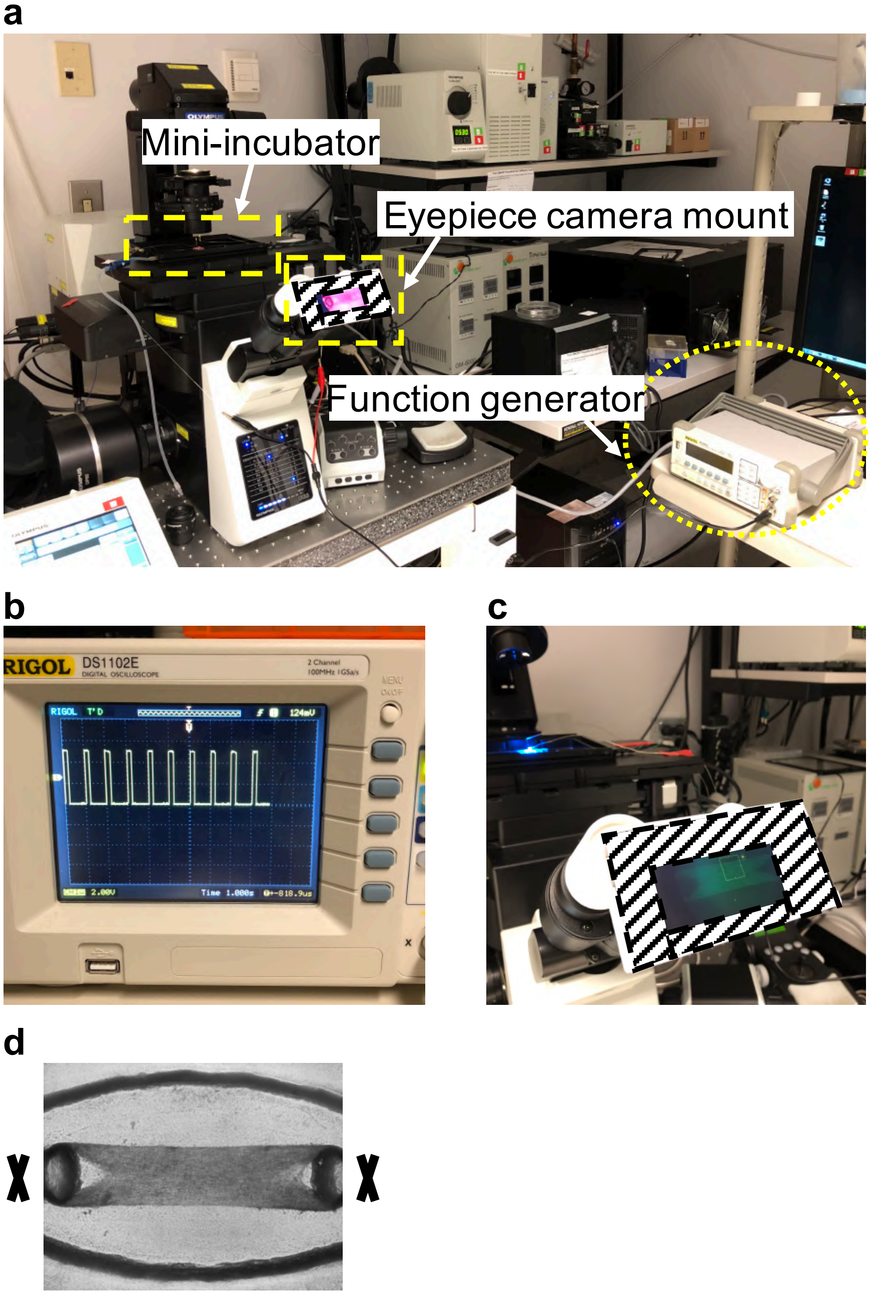

Supplementary Figure 7

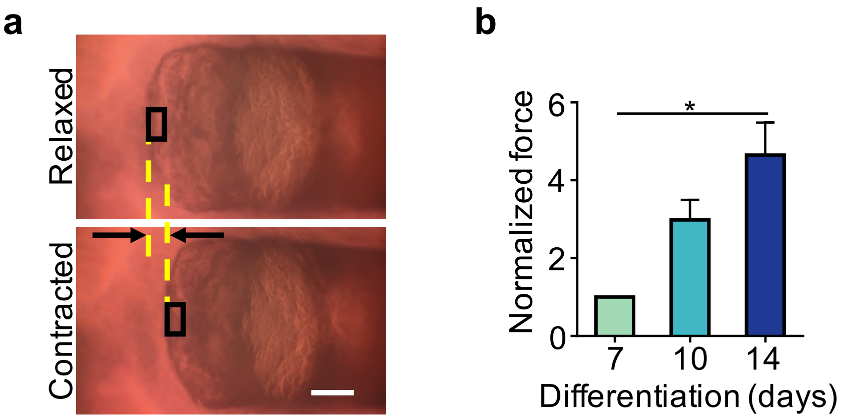

Supplementary Figure 8

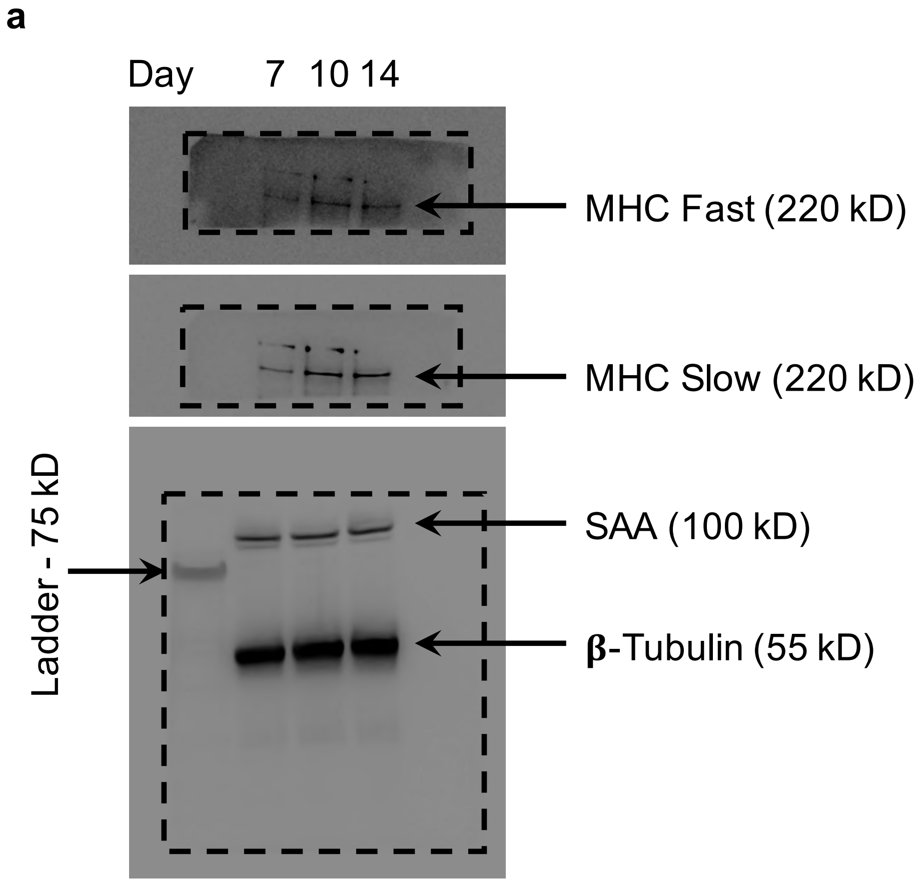

Supplemental Figure Legends

Supplemental Figure 1. MyoTACTIC three-dimensional computer aided design drawing. (a) Three-dimensional computer aided design (3D CAD) drawing of MyoTACTIC master plate. Dimensions are in mm. **(b)** Detailed drawings of the individual wells of MyoTACTIC into which the hMMTs are seeded in. Dimensions are in mm. **(c)** Total volume and surface area of the upper and lower wells, and the individual posts in each MyoTACTIC well.

Supplemental Figure 2. Primary human muscle progenitor enrichment. (a) Representative fluorescence activated cell sorting (FACS) dot plot of a primary human myoblasts culture sorted one passage following expansion of the dissociated human skeletal muscle tissue to enrich for the CD56+ fraction. A conservative final gate is applied to achieve high purity (>98%).

Supplemental Figure 3. hMMT placement on micro-posts over time and treatment. (a) Representative bright field images of MyoTACTIC well cross sections to visualize hMMT tissue position on micro-posts at differentiation Days 7, 10, and 14. Micro-posts are outlined with yellow dashed lines. The far left panel also highlights hMMT location with black dashed lines. The 'L' and 'R' labels the micro-posts on the left and right side of the well, respectively. **(b)** Dot plot indicating the distance of hMMTs from the base of micro-post pillars at Day 7, 10, and 14 of differentiation. 'L' and 'R' indicate the measured distance for the left and right post in each well respectively. n = 15 hMMTs from 3 muscle patient donors per time point and each symbol indicates data from one patient donor. No significant differences were observed between any of the experimental groups. **(c,e)** Representative bright field images of MyoTACTIC well cross sections at hMMT differentiation Day 7 and 14. hMMTs were treated with either dexamethasone **(c)**, or IGF-1 **(e)**. **(d,f)** Dot plots indicating the distance of hMMTs from the base of micro-post pillars at Day 7 and 14 of differentiation and in response to either dexamethasone **(d)**, or IGF-1 **(f)** treatments. L and R indicate the measured

distance for the left and right micro-post in each well respectively. n = 6 hMMTs from 2 muscle patient donors per time point and each symbol indicates data from one patient donor. No significant differences were observed between any of the experimental groups. **(g)** Surface roughness on the outer side of the micro-posts from a first attempt 3D printed platform (left panel) resulted in inconsistent vertical positioning of the hMMTs post remodeling (right panel). Posts are outlined with yellow dashed lines on the right panel. In **(b)**, **(d)**, and **(f)** significance was determined by two-way ANOVA followed by multiple comparisons to compare differences between groups using the Tukey's multiple comparisons test. Scale bars, 1 mm.

Supplemental Figure 4. MyoTACTIC enables bulk production of hMMTs. (a-b) Stitched bright field images of a MyoTACTIC plate **(a)** immediately following seeding with cells and extracellular matrix and before remodelling (Day -2) and **(b)** on Day 14 of differentiation. Scale bars, 10 mm.

Supplemental Figure 5. Characterization of hMMTs produced in MyoTACTIC. (a) Quantification of hMMT width over the course of culture time. n= minimum of 16 hMMTs from 3 muscle patient donors per time point. Data are presented as mean ± SEM. **(b)** Representative stitched confocal images of hMMTs counter stained with Hoechst to label nuclei (blue) and labeled for EdU (green) to visualize proliferating cells at Days 0 (top panel), 1 (middle panel), and 7 (bottom panel) of differentiation. hMMTs are outlined in white dashed lines and location of micro-posts are outlined with yellow dashed circles. Scale bar 500 μm. **(c)** Representative confocal images of the hMMTs on Day 7, 10, and 14 of differentiation immunostained for sarcomeric α-actinin (SAA, red) and α-bungarotoxin (BTX; green) to label AChR clusters. Scale bar 50 μm. **(d)** Left panel: Schematic of 3 different media change regimens. + represents replacement of all the culture media with fresh differentiation media, * indicates replacement of half of the culture media with fresh differentiation media. Right

panel: Dot plot quantification of the average myotube diameter on Day 14 of differentiation for hMMTs treated with the regimens presented in the left panel. **$p < 0.01$. Values are reported as mean ± SEM. Significance was determined by one-way ANOVA followed by multiple comparisons to compare differences between groups using Tukey's multiple comparisons test.

Supplemental Figure 6. Configuration of the electrical stimulation setup. (a) Olympus IX83 inverted microscope equipped with a fluorescence lamp was used to capture movies during electrical stimulation for post displacements and calcium handling of hMMTs. Platinum wires were hooked up to a commercial function generator (Rigol DG 1022U, outlined in yellow dashed circle on bottom right). An Apple® iPhone® SE camera in combination with a LabCam™ mount was used to capture the movies (outlined in yellow dashed box in the middle). hMMTs were kept under physiological conditions using a stage top mini incubator equipped with temperature and gas modules to control the CO2 concentrations. **(b)** A Rigol DS1102E digital oscilloscope was used to confirm the frequency and amplitude of signals before connecting the pulse generator to the platinum wires. **(c)** Representative photo of the Apple® iPhone® SE and the LabCam™ mounted on the eyepiece of the Olympus IX83 microscope. **(d)** Representative picture of an hMMT with 'X' indicating the location of the electrodes during the electrical stimulations. In (a) and (c) the trademarked aspects of the Apple® iPhone® SE are covered to remove the main identifiers of the device.

Supplemental Figure 7. Micro-post movement analysis and contractile force of hMMTs. (a) Representative bright-field images of a micro-post under 10X magnification before (relaxed) and during (contracted) a tetanus contraction using 20 Hz electrical stimuli. Yellow dashed lines and arrows indicate the displacement of the post. Scale bar 200 µm. **(b)** Bar graph quantification of the normalized contractile forces generated by hMMTs at Day 7, 10, and 14 of differentiation. Values are normalized to Day 7. *$p < 0.05$. n = minimum of 11 hMMTs from 3 muscle patient donors

per time point. Values are reported as mean ± SEM. Significance was determined by Kruskal-Wallis test followed by Dunn's multiple comparisons test to compare differences between groups.

Supplemental Figure 8. Western blot to assess contractile protein expression. (a) Representative full scan of a western blot used to generate the data in Figure 2g. Chemiluminescent signals were detected and recorded by exposure of the blots with a MicroChemi 4.2 chemiluminescence imaging system (DNR Bio-Imaging Systems). Black dashed lines indicate the borders of the blots. Images were analyzed using NIH ImageJ.

Supplemental Movie Captions

Movie S1. hMMT spontaneous contractions on Day 10 of differentiation. A series of three representative bright-field videos of hMMTs after 10 days of differentiation exhibiting spontaneous contractions. Movies were recorded using an Apple® iPhone® SE.

Movie S2. hMMTs generate twitch contractions in response to low frequency (0.5 Hz) electrical stimuli. A representative bright-field video of a hMMT generating a twitch contraction in response to low frequency (0.5 Hz) electrical stimuli. Movie was recorded using an Apple® iPhone® SE under 4X magnification.

Movie S3. hMMTs generate tetanus contractions in response to high frequency (20 Hz) electrical stimuli. A representative bright-field video of a hMMT generating a tetanus contraction in response to high frequency (20 Hz) electrical stimuli. Movie was recorded using an Apple® iPhone® SE under 4X.

Movie S4. Measurement of micro-post deflection in response to 20 Hz electrical stimulation of hMMTs using a custom Python computer vision script. A representative bright-field video of a micro-post deflection being tracked by the custom-written Python computer vision script (blue box) during 5 tetanus contractions. hMMT is stimulated using high frequency electrical stimuli (20Hz) to generate tetanus contractions. Movie was recorded using an Apple® iPhone® SE under 10X magnification and is 10X fast forwarded.

Movie S5. Micro-post deflection in response to 20 Hz electrical stimulation of hMMTs on Day 7, 10, and 14 of differentiation. A series of three bright-field representative movies of micro-post deflection in response to hMMT tetanus contractions on Days 7, 10, and 14 of differentiation. hMMTs are stimulated using high frequency electrical stimuli (20Hz) to generate tetanus contractions. Movies were recorded using an Apple® iPhone® SE under 4X magnification and are 3X fast forwarded.

Movie S6. Spontaneous calcium handling of hMMTs on Day 7 differentiation. A representative epifluorescence time-lapse video of hMMTs after 7 days of culture demonstrating spontaneous calcium transients. Calcium transients are visualized in green by following the GCaMP6 calcium reporter signal that was transduced into the human muscle cells.

Movie S7. hMMT twitch and tetanus contraction calcium handling on differentiation Days 7, 10, and 14. A series of three representative videos of hMMTs after 7, 10, and 14 days of differentiation stimulated with 0.5 Hz electrical stimuli. Myotube calcium transients are visualized in green by following the GCaMP6 calcium reporter signal that was transduced into the human muscle cells. Movies are fast forwarded 3X.

Movie S8. hMMT calcium handling on differentiation Days 7, 10, and 14 in response to biochemical stimuli. A series of three representative videos of hMMTs after 7, 10, and 14 days of differentiation and stimulated with ACh (2mM). Myotube calcium transients are visualized in green by following a GCaMP6 calcium reporter signal that was transduced into the human muscle cells. Movies are fast forwarded 3X.

Movie S9. d-tubocurarine treatment blocks hMMT response to ACh but has no effect on hMMT response to electrical stimuli.

A series of three representative videos of a hMMTs on Day 14 of differentiation following treatment with d-tubocurarine (25 μM) and stimulation with electrical (0.5 Hz and 20Hz) and biochemical (ACh, 2mM) stimuli. Myotube calcium transients are visualized in green by following a GCaMP6 calcium reporter that was transduced into the human muscle cells. Movies are fast forwarded 3X.

Movie S10. Dexamethasone treatment reduces the contractile force generation of hMMTs compared to control (untreated) hMMTs as indicated by post deflection analysis. A series of two bright-field representative movies of micro-post deflection in response to hMMT tetanus contractions on Day 14 of differentiation. hMMTs are treated with vehicle (DMSO, control) or Dexamethasone (10 nM) from Day 7 to day 14 of differentiation. hMMTs are stimulated using high frequency electrical stimuli (20Hz) to generate tetanus contractions. Movies were recorded using an Apple® iPhone® SE under 4X magnification and are 6X fast forwarded.

Movie S11. Cerivastatin treatment reduces the contractile force generation of hMMTs compared to control (untreated) hMMTs as indicated by post deflection analysis. A series of two bright-field representative movies of micro-post deflection in response to hMMT tetanus contractions on Day 14 of differentiation. hMMTs are treated with vehicle (DMSO, control) or Cerivastatin (10 nM) from Day 7 to 14 of differentiation. hMMTs are stimulated using high frequency electrical stimuli (20Hz) to generate tetanus contractions. Movies were recorded using an Apple® iPhone® SE under 10X magnification and are 6X fast forwarded.

Movie S12. IGF-1 treatment increases the contractile force generation of hMMTs compared to control (untreated) hMMTs as indicated by post deflection analysis. A series of two bright-field representative movies of micro-post deflection in response to hMMT tetanus contractions on Day 14 of differentiation. hMMTs are treated with vehicle (DMEM, control) or IGF-1 (100 nM) from Day 7 to 14 of differentiation. hMMTs are stimulated using high frequency electrical stimuli (20Hz) to generate tetanus contractions. Movies were recorded using an Apple® iPhone® SE under 10X magnification and are 6X fast forwarded.

Movie S13. Gemcitabine treatment of hMMTs at supraphysiological dose does not affect their contractile force generation compared to control (untreated) hMMTs. A series of two bright-field representative movies of micro-post deflection in response to hMMT

tetanus contractions on Day 14 of differentiation. hMMTs are treated with one-time dose of vehicle (DMSO, control) or Gemcitabine (320 nM) on Day 7 of differentiation. hMMTs are stimulated using high frequency electrical stimuli (20Hz) to generate tetanus contractions. Movies were recorded using an Apple® iPhone® SE under 10X magnification and are 6X fast forwarded.

Movie S14. Irinotecan treatment reduces the contractile force generation of hMMTs compared to control (untreated) hMMTs as indicated by post deflection analysis. A series of three bright-field representative movies of micro-post deflection in response to hMMT tetanus contractions on Day 14 of differentiation. hMMTs are treated with one-time dose of vehicle (DMSO, control) or Irinotecan (16 nM, 72 nM) on Day 7 of differentiation. hMMTs are stimulated using high frequency electrical stimuli (20Hz) to generate tetanus contractions. Movies were recorded using an Apple® iPhone® SE under 10X magnification and are 6X fast forwarded.

Movie S15. Measurement of force-displacement relation of MyoTACTIC micro-posts using Microsquisher. Representative movie demonstrating the displacement of the micro-posts in response to the force exerted by the micro-wire connected to a force transduce to generate a force-displacement correlation for micro-post deflection. Location of the post and the micro-wire are indicated with black arrows.

Movie S16. ROIs for calcium transients and contractile force analyses. A bright-field movie of a hMMT under high frequency electrical stimulation (20Hz) captured with an Apple® iPhone® SE demonstrating various regions of interests for data collection and analysis.

Table S1. List of primary antibodies

#	Antibody	Species	Dilution	Vendor (CAT-Nr.)
1	Alexa Fluor 647 mouse anti-human CD56	Mouse	1:20	BD Pharmingen (557711)
2	Anti- β -tubulin	Rabbit	1:5000	Cell Signaling (2146)
3	DRAQ5	-	1:1000	abcam (ab108410)
4	Hoechst 33342	-	1:1000	ThermoFisher (H3570)
6	Myosin heavy chain - fast	Mouse	1:50	DSHB (A4.74)
7	Myosin heavy chain - slow	Mouse	1:50	DSHB (A4.951)
8	Sarcomeric α-actinin	Mouse	1:800 (IF)<br>1:2000 (WB)	Sigma (A7811)
9	α-Bungarotoxin, Alexa Fluor 647 conjugate	-	1:500	ThermoFisher (B35450)

Table S2. Cell Culture Media and Solutions

#	Name	Details
1	Blocking solution	20 % goat serum, 0.3 % Triton-X 100 in PBS
2	Fibrinogen stock solution	10 mg / mL fibrinogen in 0.9 % (wt / v) NaCl solution in water
3	Human myoblast differentiation media	Dulbecco's Modified Eagle's medium (DMEM), 2 % horse serum, 10 µg / mL insulin, 1% penicillin-streptomycin
4	Human myoblast growth media	Ham's F-10 nutrient mix, 20 % fetal bovine serum, 5 ng / mL basic fibroblast growth factor, 1 % penicillin-streptomycin
5	Hydrogel mixture	Dulbecco's Modified Eagle's medium (DMEM) (40% v/v), 4 mg / mL bovine fibrinogen (40% v/v), Geltrex^{TM} (20% v/v), thrombin (0.2 unit / mg fibrinogen)
6	Milk based blocking solution	5 % (wt / v) skim milk (BioShop) in TBST
7	Red blood cell lysis buffer	15.5 mM NH4Cl, 1 mM KHCO3, 10 µM EDTA
8	Tris-buffered saline Tween (TBST)	50 mM Tris (BioShop), 150 mM NaCl (Sigma), 0.1 % (v / v) Tween 20 (BioShop)

Table S3. List of drugs

#	Name	Vendor (Product #)
1	Cerivastatin	Sigma (SML0005)
2	Dexamethasone	Sigma (D1756)
3	Gemcitabine	Sigma (G6423)
4	IGF-1	Sigma (I1271)
5	Irinotecan	Cayman Chemical Company (14180)

Table S4. MyoTACTIC production success rate from 3D printed molds

# of usable wells in the hard 3D printed platform	# of usable wells in the PU Mold	Success rate (step 1 - 3)	# of usable wells in the PDMS platform	Success rate (step 4)	# of platforms casted from the PU Mold
91	81	89.0 %	81	100%	> 100
91	84	92.3 %	84	100%	> 100

Table S5. Primary human myoblast patient donor information

STEM LINE	AGE	SEX	SURGERY LOCATION
21	64	Female	Lumbar
32	60	Male	Lumbar
38	68	Male	Lumbar
46	64	Female	Lumbar
50	51	Male	Lumbar
86	60	Male	Lumbar

Appendix

Post tracking Python code

This computer vision script, when run, prompts the user to select a file containing the video of interest and select the region of interest on the MyoTACTIC post. The script then tracks the horizontal position of the post in pixels over the course of the video and calculates the displacements associated with human muscle microtissue contractions. For the experiments described here, the MyoTACTIC post edge farthest from the tissue was chosen as the region of interest. However other users may find different regions of interest to be more optimal for their own purposes. This post tracking script is written in Python 3, and requires the matplotlib library and the opencv-contrib library to run. This script has been tested on Windows 10 Home and macOS Version 10.14.2 operating systems.

The below presented script can track the horizontal position of the post to the extent where the region of interest (black rectangle in Supplementary Figure 7a) is not obscured by the environmental factors such as background.

```python
from tkinter import filedialog
import cv2
import sys
import matplotlib
matplotlib.use("TkAgg")
import matplotlib.pyplot as plt


class ClickLocation:

    def __init__(self):
        self.roi_center = None

    def click(self, event, x, y, flags, param):
        if event == cv2.EVENT_LBUTTONDOWN:
            self.roi_center = (x,y)


vid_path = filedialog.askopenfilename(
        initialdir="C:/",
        filetypes=(("All Files","*.*"), ("Text File", "*.txt")),
        title= "Choose a file.")

contraction_vid = cv2.VideoCapture(vid_path)
colour = (255,50,50)
frame_counter = 1
user_roi = False
```

```python
gray_history = []
fx = 0.55
fy= 0.55


ok, first_frame = contraction_vid.read()
first_frame_gray = cv2.cvtColor(first_frame, cv2.COLOR_BGR2GRAY)
first_frame_gray = cv2.equalizeHist(first_frame_gray)

if not ok:
    print("Cannot read first frame")
    sys.exit()

'''''
Due to screen size constraints, images shown to user are all resized versions
of the frames that the tracking is done on. ROI format is:
(top_left_corner_x_coord, top_left_corner_y_coord, ROI_width, ROI_height).
'''
#ROI width/height factors. Adjust if necessary.

#larger ROI - less sensitive but better able to track very large deflections
#Smaller ROI - more sensitive, better able to track smaller deflections

#RHF = 0.25
#RWF = 0.08

#RHF = 0.2
#RWF = 0.06

#Default ROI size
RHF = 0.1
RWF = 0.03

resized_frame = cv2.resize(first_frame, (0,0), fx=fx, fy=fy)

cv2.namedWindow("Identify post")
clickLocation = ClickLocation()
cv2.setMouseCallback("Identify post", clickLocation.click)

while (True):

    marked_frame = resized_frame.copy()
    if not clickLocation.roi_center is None:
        cv2.circle(marked_frame,(clickLocation.roi_center[0],
                                 clickLocation.roi_center[1]), 5,(255,0,0),1)

        #Get user click location, use to draw ROI
        roi_center = clickLocation.roi_center

        user_top_left = (int(roi_center[0] -
                             round(RWF * resized_frame.shape[1]/2)),
        int(roi_center[1] - round(RHF * resized_frame.shape[0]/2)))

        user_bottom_right = (int(roi_center[0] +
                                 round(RWF * resized_frame.shape[1]/2)),
        int(roi_center[1] + round(RHF * resized_frame.shape[0]/2)))

        user_roi = (user_top_left[0], user_top_left[1],
                    user_bottom_right[0] - user_top_left[0],
                    user_bottom_right[1] - user_top_left[1])

        cv2.rectangle(marked_frame, (user_top_left), (user_bottom_right),
                      colour, 1)
    cv2.imshow("Identify post", marked_frame)

    h = cv2.waitKey(1)
```

```python
    if h & 0xFF == ord('\r'):
        break

roi = (round(user_roi[0]/fx), round(user_roi[1]/fy),
       round(user_roi[2]/fx), round(user_roi[3]/fy))

top_left = (roi[0], roi[1])
bottom_right = (roi[0] + roi[2], roi[1] + roi[3])

#Draw rectangle. Rectangle takes top left and bottom right points as params
cv2.rectangle(resized_frame, (user_top_left), (user_bottom_right), colour, 1)
cv2.imshow("Frame", resized_frame)
cv2.moveWindow("Frame", 0,0)

cv2.waitKey(0)
cv2.destroyWindow("Frame")

tracker = cv2.TrackerKCF_create()

#Initialize tracker with first frame
tracker.init(first_frame_gray, roi)
gray_history.append(first_frame_gray)

#log x-axis location of post, i.e. center of roi
post_location = [roi[0] + roi[2]/2]

#Go to second frame
unfinished, frame = contraction_vid.read()

while unfinished:

    frame_gray = cv2.cvtColor(frame, cv2.COLOR_BGR2GRAY)
    frame_gray = cv2.equalizeHist(frame_gray)
    resized_frame = cv2.resize(frame, (0,0), fx=fx, fy=fy)
    tracked, roi = tracker.update(frame_gray)
    gray_history.append(frame_gray)

    if tracked:
        #Tracker has identified post

        #Roi points are floating point, must cast to int

        top_left = (int(roi[0]), int(roi[1]))
        bottom_right = (int(roi[0]) + int(roi[2]), int(roi[1]) + int(roi[3]))

        resized_top_left = (round(top_left[0]*fx),round(top_left[1]*fy))
        resized_bottom_right =  (resized_top_left[0] + round(roi[2]*fx),
                                 resized_top_left[1] + round(roi[3]*fy))

        #Resize roi for depicting w/ imshow to fit screen
        cv2.rectangle(resized_frame, resized_top_left,resized_bottom_right,
                      colour, 1)

        cv2.imshow("Frame",resized_frame)
        cv2.waitKey(1)

        #log x-axis location of post
        post_location.append(top_left[0] + roi[2]/2)
    else:

        #If tracking failed, roi becomes = (0.0, 0.0, 0.0, 0.0)

        cv2.putText(resized_frame, "TRACKER FAILED",
                    (resized_frame.shape[1]//2, resized_frame.shape[0]//2),
                    cv2.FONT_HERSHEY_SIMPLEX, 0.75, (0,0,0), 2)

```

```
        print("TRACKER FAILED AT FRAME {}".format(frame_counter))
        cv2.imshow("Frame",resized_frame)
        cv2.waitKey(1)

        #Store aberrant post_location
        post_location.append(top_left[0])

    unfinished, frame = contraction_vid.read()
    frame_counter += 1


cv2.destroyAllWindows()

#From post locations determine if post is contracting to 'right' or 'left'
#i.e. in direction of increasing or decreasing pixel location.'''

rightmost = max(post_location)
leftmost = min(post_location)
normalized_post_location = []

for i in post_location:

    normalized_post_location.append(abs(post_location[0] - i))

#Assuming video starts with relaxed tissue, post will be farthest from initial
#location during a contraction. If post contracts to right,
#the difference between rightmost and the initial post location will be much
#greater than the difference between leftmost and the intial post location
#. And vice-versa if post contracts to the left.

if rightmost - post_location[0] > post_location[0] - leftmost:
    contracts_to_right = True
elif post_location[0] - leftmost > rightmost - post_location[0]:
    contracts_to_right = False
else:
    print("Unable to determine direction of post movement")
    contracts_to_right = True

multiple_contractions = input("Multiple contraction video? [Y/N] ")
max_displacement = -1
displacements = []

#If tissue contracts to right, maxima = contractions, minima = relaxations.
#If tissue contracts to left, maxima = relaxations and minima = contractions.
maxima_indices = []
minima_indices = []

#Error factor for identifying local maxima and minima. Adjust if necessary
error = 2

if multiple_contractions.upper() == 'N':

    max_displacement = abs(max(post_location) - min(post_location))
    print("Contraction displacement = {} pixels.".format(max_displacement))


else:

    mins = [post_location[0]]
    minima_indices.append(0)
    maxes = []

    contracting = True

    if contracts_to_right:

```

```
        #Initialize running max/min to values which will pass first test
        running_max = -1
        running_min = 1E6

        for i in range(len(post_location)):

            if i + 3 >= len(post_location):
                break

            #Store next three post locations
            next1, next2, next3 = (post_location[i + 1],
            post_location[i + 2], post_location[i + 3])

            if contracting:

                if post_location[i] >= running_max:
                    running_max = post_location[i]
                    running_max_index = i

                #If tissue has begun relaxing/stopped contracting, store
                #running_max as true local maximum. Error margin included in
                #case of bbox drift.

                if (max([next1, next2, next3, running_max]) == running_max and
                running_max - min([next1, next2, next3])) >= error:
                    contracting = False
                    maxes.append(post_location[running_max_index])
                    maxima_indices.append(running_max_index)
                    running_min = 1E6

            elif not contracting:

                if post_location[i] <= running_min:
                    running_min = post_location[i]
                    running_min_index = i


                #If tissue has begun contracting/stopped relaxing, store
                #running_min as true local maximum. Error margin included in
                #case of bbox drift.

                if (min([next1, next2, next3, running_min]) == running_min and
                max([next1, next2, next3]) - running_min) >= error:
                    contracting = True
                    mins.append(post_location[running_min_index])
                    minima_indices.append(running_min_index)
                    running_max = -1


    #If contracts to left, post location will decrease with contraction
    elif not contracts_to_right:


        #Initialize running max/min to values which will pass first test
        running_max = 1E6
        running_min = -1

        for i in range(len(post_location)):

            if i + 3 >= len(post_location):
                break

            #Store next three post locations
            next1, next2, next3 = (post_location[i + 1],
            post_location[i + 2], post_location[i + 3])

```

```python
            if contracting:

                if post_location[i] <= running_max:
                    running_max = post_location[i]
                    running_max_index = i

                #If tissue has begun relaxing/stopped contracting, store
                #running_max as true local maximum. Error margin included in
                #case of bbox drift.

                if (min([next1, next2, next3, running_max]) == running_max and
                max([next1, next2, next3]) - running_max >= error):
                    contracting = False
                    maxes.append(post_location[running_max_index])
                    maxima_indices.append(running_max_index)
                    running_min = -1

            elif not contracting:

                if post_location[i] >= running_min:
                    running_min = post_location[i]
                    running_min_index = i


                #If tissue has begun contracting/stopped relaxing, store
                #running_min as true local maximum. Error margin included in
                #case of bbox drift.

                if (max([next1, next2, next3, running_min]) == running_min and
                running_min - min([next1, next2, next3])) >= error:
                    contracting = True
                    mins.append(post_location[running_min_index])
                    minima_indices.append(running_min_index)
                    running_max = 1E6


    for i in range(len(maxes)):

        #Default: displacement = contraction - most recent relaxation.
        # This was used for all hMMT experiments.

        displacements.append(abs(maxes[i] - mins[i]))

        #Alternative: displacement = contraction - initial relaxed state
        #displacements.append(abs(maxes[i] - mins[0]))

    output = ('''Maxes: {}, Mins: {}\nRelative displacements: {}\nContracted to right: {}'''
              .format(maxes, mins, displacements, contracts_to_right))

    extra_output = ""

    if multiple_contractions.upper() == 'BOTH':
        max_displacement = abs(max(post_location) - min(post_location))

        extra_output = '''\nIf single video method, contraction displacement =
        {} pixels.'''.format(max_displacement)

    output = output + extra_output

    out_file = open("postTracking.txt", "w")
    out_file.write(output)
    out_file.close()

    print(output)
```

```
plt.plot(normalized_post_location)
plt.show()
plt.pause(0.001)
#If running from Command Prompt/Terminal, close all GUI windows to progress

export = input("Export post locations as .csv file? [Y/N] ")

if export.upper() == "Y":
    csv_file = open("postTracking.csv", "w")
    csv_locations = ""
    csv_locations += "Normalized post locations, Raw post locations,\n"
    for i in enumerate(normalized_post_location):
        csv_locations += str(i[1]) + "," + str(post_location[i[0]]) + ",\n"

    csv_file.write(csv_locations)
    csv_file.close()
```
